# Synthesis and structures of anionic rhenium polyhydride complexes of boron–hydride ligands and their application in catalysis†‡

**DOI:** 10.1039/d0sc03458d

**Published:** 2020-09-09

**Authors:** Liam J. Donnelly, Simon Parsons, Carole A. Morrison, Stephen P. Thomas, Jason B. Love

**Affiliations:** EaStCHEM School of Chemistry, University of Edinburgh Joseph Black Building, David Brewster Road, The King's Buildings Edinburgh EH9 3FJ UK jason.love@ed.ac.uk stephen.thomas@ed.ac.uk

## Abstract

The rhenium complex, [K(DME)(18-c-6)][ReH_4_(Bpin)(η^2^-HBpin)(κ^2^-H_2_Bpin)] **1**, comprising hydride and boron ligands only, has been synthesized by exhaustive deoxygenation of the commercially available perrhenate anion (ReO_4_^−^) with pinacol borane (HBpin). The structure of **1** was analysed by X-ray crystallography, NMR spectroscopy, and DFT calculations. While no hydrides were located in the X-ray crystal structure, it revealed a trigonal arrangement of pinacol boron ligands. Variable-temperature NMR spectroscopy supported the presence of seven hydride ligands but further insight was hindered by the fluxionality of both hydride and boron ligands at low temperature. Further evaluation of the structure by *Ab Initio* Random Structure Searching (AIRSS) identified the presence of hydride, boryl, σ-borane, and dihydroborate ligands. This complex, either isolated or prepared *in situ*, is a catalyst for the 1,4-hydroboration of N-heteroaromatic substrates under simple operating procedures. It also acts as a reagent for the stoichiometric C–H borylation of toluene, displaying high *meta* regioselectivity in the borylated products. Reaction of **1** with 9-BBN resulted in HBpin substitution to form the new anionic tetra(dihydroborate) complex [K(DME)(18-c-6)][Re(κ^2^-H-9-BBN)_4_] **4** for which the hydride positions were clearly identified by X-ray crystallography. The method used to generate these isolable yet reactive boron–hydride complexes is direct and straightforward and has potential utility for the exploitation of other metal oxo compounds in operationally simple catalytic reactions.

## Abstract 2

Exhaustive deoxygenation of perrhenate by pinacol borane forms a new Re anion of boron and hydride ligands only that undergoes borane ligand exchange, stoichiometric C–H boration, and catalytic pyridine hydroboration.

## Graphical abstract

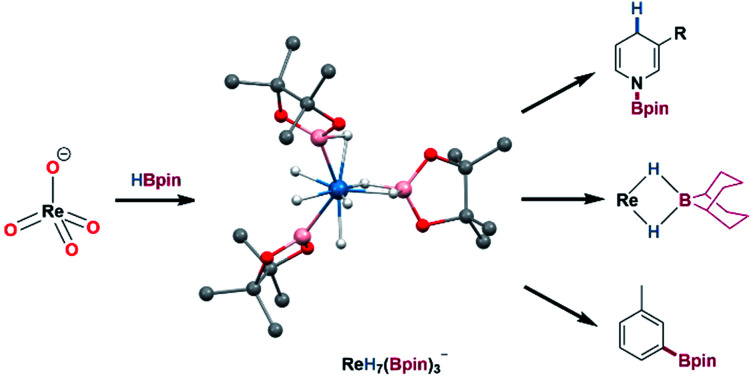

## Introduction

**Scheme 1:**
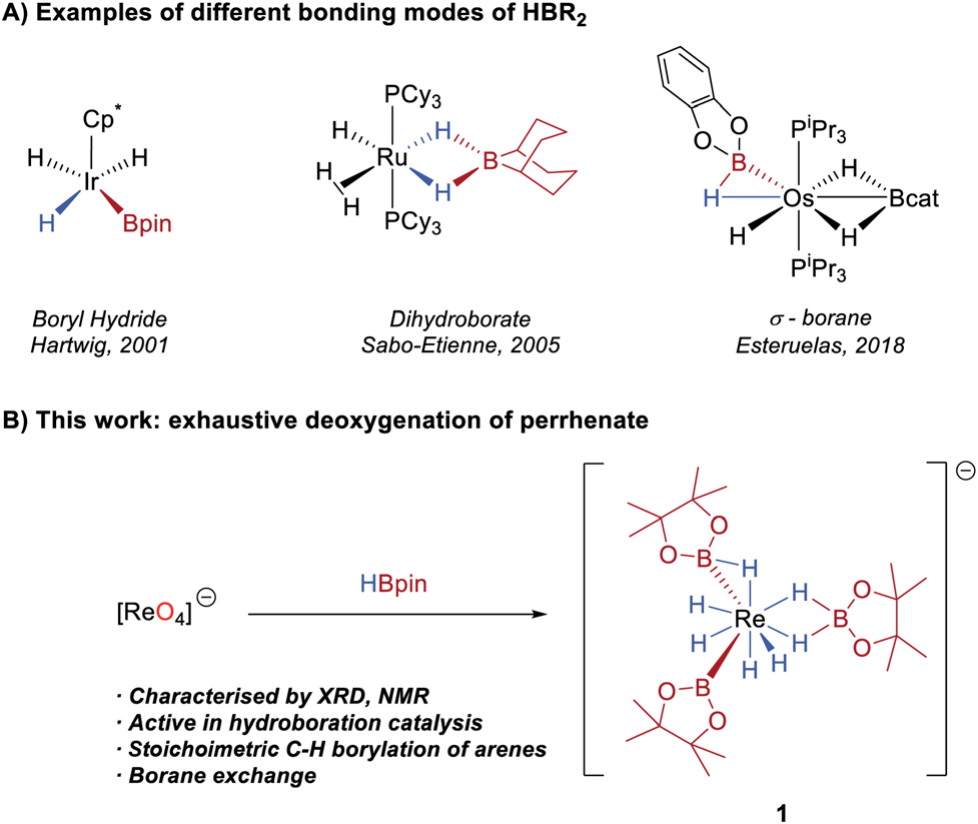
(A) Selected examples of complexes with boron-centred ligands. (B) This work: synthesis of a rhenium boron–polyhydride anion from HBpin and KReO_4_.

The activation of a B–H bond by a transition-metal complex is a key step in catalytic processes such as the borylation of organic molecules^1^ and the dehydrocoupling of ammonia-borane.^2^ As such, the synthesis and study of boron–hydride complexes is an area of growing interest.^3^ A number of studies have shown that hydroboranes can adopt different coordination geometries at a metal centre, a feature which is largely determined by the Lewis acidity of the borane and the Lewis basicity of the metal (Scheme 1A). At one extreme of the bonding continuum are metal boryl hydrides resulting from oxidative addition of the B–H bond to the metal centre, exemplified in Rh,^4^ Ir,^4*a*,5^ Os,^6^ Ru,^4*d*^ Nb,^7^ Ta^7*a*^ and W complexes.^7*b*,8^ At the other extreme, the boron ligand coordinates as a dihydroborate, and which is typically observed for more Lewis-acidic boranes such as 9-borabicyclo[3.3.1]nonane (9-BBN).^6,9^ Lying between these two extremes are a number of potential *σ*(B–H) complexes.^5*b*,9*g*,10^

High oxidation-state metal-oxo complexes have been studied in the activation of X–H bonds (X = B, Si, P) for application in catalytic hydrofunctionalisation reactions of unsaturated organic substrates and in deoxydehydration reactions, both of which represent a role-reversal from their traditional use in oxidation chemistry.^11^ Recently, we reported that [N(hexyl)_4_][ReO_4_] can act as a catalyst for the reduction of organic carbonyls and carbon dioxide to the corresponding alcohols by activation of hydrosilanes.^12^ Similarly, the reduction of sulfoxides by pinacol borane (HBpin) and various Re-oxo catalysts has been reported to proceed by addition of the B–H bond across a Re=O bond to form a boroxy–hydride complex.^13^

We became interested in exploring the use of the perrhenate anion [ReO_4_]^−^, the most commonly traded form of Re,^14^ as a synthetic precursor for rhenium boron–hydride complexes through the activation of the B–H bonds of common boranes, *e.g.* HBpin (pin = pinacol). This contrasts with the normal synthetic routes to these types of complexes, which typically involve salt metathesis reactions with an electrophilic boron reagent^15^ or by reaction of a nucleophilic boryl with a rhenium halide;^16^ these methods require the use of pre-functionalized and air-sensitive reagents and are limited to low oxidation-state Re centres. Furthermore, rhenium (poly)hydrides are typically synthesised from Re oxides under forcing conditions such as sodium in EtOH^17^ or by the reaction of a rhenium (oxo)halide with a main group metal hydride such as LiAlH_4_.^18^

Herein, we report the operationally simple synthesis of [K(DME)(18-c-6)][ReH_4_(Bpin)(η^2^-HBpin)(κ^2^-H_2_Bpin)] **1** through the exhaustive deoxygenation of KReO_4_ by HBpin (Scheme 1B). This complex comprises hydride, boryl, σ-borane, and dihydroborate ligands and is an active catalyst for the 1,4-hydroboration of N-heteroaromatics. A protocol has been developed such that the simple, air-stable alklyammonium complexes [NR_4_][ReO_4_] can be exploited as pre-catalysts for hydroboration reactions. We also show that **1** can be further functionalized through borane exchange and is a reagent for stoichiometric and regioselective C–H borylation of arenes.

## Results and discussion

### Synthesis and structural characterisation of **1**

**Scheme 2:**
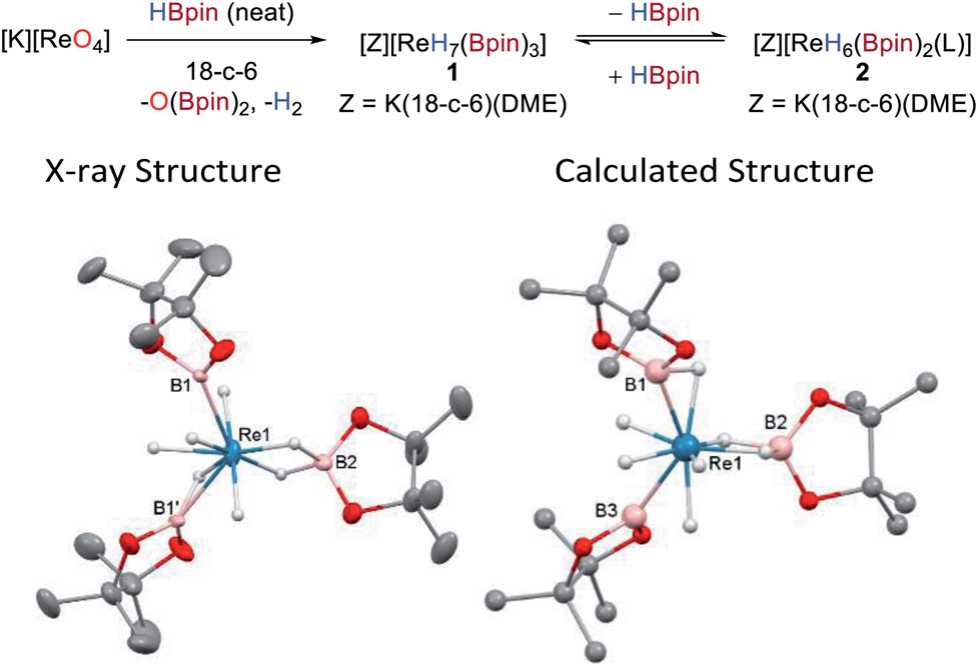
Synthesis of **1** from [K][ReO_4_] (L = THF) and the solid-state (left) and calculated (right) structures of **1**. For clarity, the cation [K(DME)(18-c-6)]^+^ and all hydrogen atoms except for the Re hydrides are omitted from the X-ray crystal structure; displacement ellipsoids are drawn at 50% probability.

The reaction between KReO_4_ and HBpin in the presence of 18-crown-6 (18-c-6) forms the new boron polyhydride complex [K(18-c-6)_2_][ReH_7_(Bpin)_3_], O(Bpin)_2_, and H_2_ as a result of the complete deoxygenation of KReO_4_. Recrystallization of the reaction mixture from toluene/DME allows isolation of the product [K(DME)(18-c-6)][ReH_4_(Bpin)(η^2^-HBpin)(κ^2^-H_2_Bpin)] **1** in 56% yield as colourless needles (Scheme 2). The same anionic moiety is observed by ^1^H and ^11^B NMR spectroscopy when perrhenates of alkylammonium countercations, *e.g.* NBu_4_^+^ or N(hexyl)_4_^+^ are used instead of potassium (Fig. S8 and S9‡); however, in these former cases the isolation of the deoxygenated product is more challenging due to their enhanced solubility in common organic solvents. The ^1^H NMR spectrum of **1** in d_8_-toluene exhibits a singlet hydride resonance at −7.22 ppm integrating to 7 hydrogens which remains sharp on cooling to 173 K. The ^11^B NMR spectrum shows a single broad resonance at 45.6 ppm which, similarly to the ^1^H NMR resonances, is unchanged at low temperature. The IR spectrum of **1** shows weak and broad absorptions that are attributable to Re–H stretches between 2000 and 1750 cm^−1^. Single-crystal X-ray diffraction was carried out on a two-domain aggregate crystal of **1** from which diffraction images were integrated for both domains using two orientation matrices. The solid-state structure shows that two of the Bpin ligands (B1, B1′) are related by a *C*_2_-symmetry axis and, along with B2 adopt a pseudo-trigonal arrangement at the Re atom (B1–Re1–B2 = 118.6(2)°, B1–Re1–B1′ = 122.9(5)°) with similar Re1–B1 (2.198(10) Å) and Re1–B2 (2.174(14) Å) distances (Scheme 2, left); these distances compare well with other high oxidation-state boron–hydride complexes.^4*a*–*c*,5*a*,7*b*,19^

The hydrides in **1** could not be located with confidence from difference maps. As such, possible hydride positions were identified using *ab initio* random structure searching (AIRSS, see ESI‡ for details) with the positions of the heavier atoms derived from the crystal structure and constraints on prospective hydride positions to ensure neither H_2_ was formed nor unwanted K–H interactions occurred.^20^ This approach generated a series of structures with various combinations of hydride, boryl, σ-borane, and hydroborate ligands (Fig. S36‡). Higher energy structures featuring two boryl or three dihydroborate ligands were discounted, leaving a series of closely related, low-energy structures (within 5 kJ mol^−1^) comprising three different binding modes for the boron ligands, a dihydroborate (B2), a σ-borane (B1), and a boryl (B3), along with four terminal hydrides (Scheme 2, right). This model was then used to place the hydrides in the crystal structure refinement (Scheme 2, left, see ESI‡ for details); while refinement is successful there is only a very minor enhancement to its quality. The geometry optimized and crystal structures show significant differences in the Re1–B2 distances (calcd 2.334 Å *cf.* X-ray 2.175 Å) and the B1–Re1–B1′/B3 angles (calcd 130.2° *cf.* X-ray 122.9°) which might arise from disorder in the crystal structure of the anion such that its geometrical parameters are averages (see ESI‡ for details). Also, the calculated structure portrays a formal Re(v) oxidation state, whereas the short Re–B distances in the crystal structure and the ^11^B NMR data are consistent with more boryl character and suggest a Re(vii) oxidation state. It is therefore evident that rapid hydride/boron ligand rearrangement occurs as seen with other transition metal hydrides,^5*a*,*c*^ potentially by a σ-CAM mechanism,^21^ resulting in a range of energetically similar structures.^22^ This uncertainty renders the formulation of **1** with respect to the hydride positions as tentative.

Dissolution of **1** in d_8_-THF results in a different ^1^H NMR spectrum to that seen in d_8_-toluene, showing a major signal at −7.74 ppm and a minor signal at −7.32 ppm that slightly increases in intensity over the course of 72 h (Fig. S4 and S5‡). This feature is concomitant with the formation of free HBpin and the growth of a second signal at 41.9 ppm in the ^11^B NMR spectrum. These spectra suggest that an equivalent of HBpin dissociates upon dissolution of **1** in donor solvents to form the related rhenium boron–polyhydride, [K(DME)(18-c-6)][ReH_6_(Bpin)_2_(L)] **2**, where L in this instance is THF; decomposition of the two Re species to an insoluble material also occurs over time (see below). This ease of ligand exchange may be important in terms of the catalytic activity of **1**.

### Stoichiometric reactivity of **1**

**Scheme 3:**
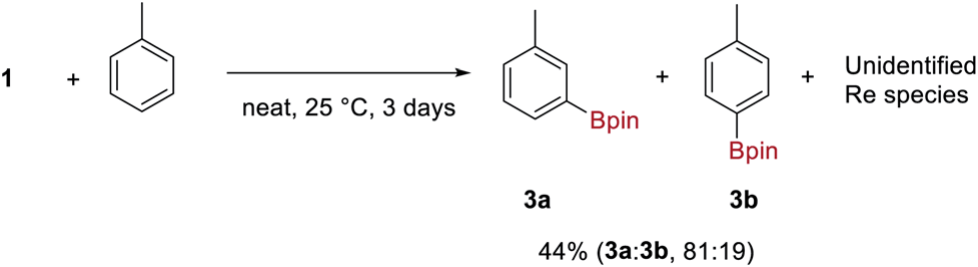
Stoichiometric borylation of toluene by **1**. Regioselectivity and yields were determined by ^1^H NMR spectroscopy from the crude reaction mixture using trimethoxybenzene as an internal standard and averaged over 3 runs.

Complex **1** is indefinitely stable in the solid-state when stored under N_2_ at −20 °C but decomposes with loss of H_2_ and HBpin over 3–5 days in ethereal solvents at room temperature to give an insoluble and unidentified dark-brown solid. In contrast, **1** reacts cleanly with aromatic solvents such as C_6_H_6_ and toluene over 3 days at room temperature to form the aryl boronic esters **3** and an insoluble, dark-brown rhenium species. Toluene undergoes C–H borylation with high regioselectivity for the *meta* C–H over the *para* C–H (81:19) (Scheme 3). To the best of our knowledge, this is the highest reported *meta* selectivity observed for the stoichiometric or catalytic C–H borylation of toluene.^23^ This reaction is likely to proceed by a similar mechanism reported for [Ir(Bpin)_3_(L)_2_] complexes (where L is a bisphosphine, bipyridine or phenanthroline ligand) which undergo σ-CAM activation of the arene C–H bond.^24^ This is the first example of a high oxidation-state rhenium complex mediating C–H borylation, contrasting with previous examples that exploit complexes of lower oxidation-states.^25^

**Scheme 4:**
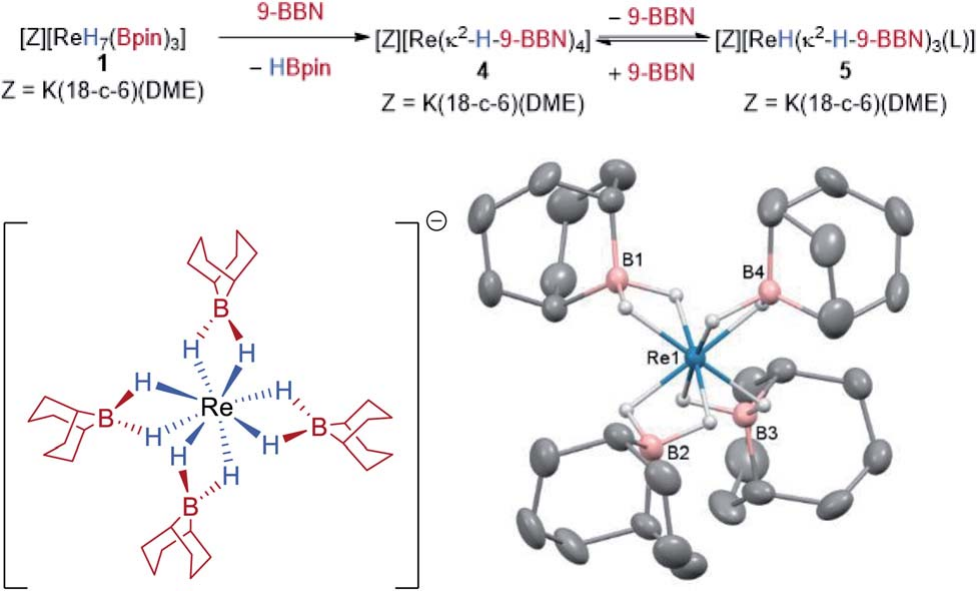
Synthesis of **4** from **1** by borane exchange and the solid-state structure of **4**. For clarity, the cation [K(DME)(18-c-6)]^+^ and all hydrogen atoms except for the Re hydrides are omitted; displacement ellipsoids are drawn at 50% probability.

In addition to C–H functionalization, **1** also undergoes borane exchange. Reaction of **1** with 2 equivalents of 9-BBN dimer (9-BBN = 9-borabicyclo[3.3.1]nonane, HBR_2_) forms [K(DME)(18-c-6)][Re(κ^2^-H-9-BBN)_4_] **4**, along with three equivalents of HBpin (Scheme 4). The solid-state structure of **4** reveals dihydroborate ligands in a tetrahedral geometry at the Re atom (average B–Re–B = 109.1°). The hydrides were located in the difference Fourier map and display an average B–H bond length of 1.284(2) Å, with the average Re–B distance of 2.296(2) Å similar to those seen in the optimized structure of **1** and to related dihydroborate complexes.^9*f*,19,26^ The IR spectrum of **4** does not display any absorptions attributable to Re–H stretches which further supports a borohydride bonding motif. Complex **4** is insoluble in aromatic and alkane solvents. On dissolution in d_8_-THF, the ^1^H NMR spectrum of **4** shows two hydride signals at −9.02 ppm (s, br) and −9.80 ppm (s) which have a combined integration of 8 protons. As free 9-BBN monomer is seen in the ^11^B NMR spectrum, it is evident that substitution of 9-BBN by THF occurs to form a mixture of **4** and presumably the THF-adduct [K(DME)(18-c-6)][ReH(κ^2^-H-9-BBN)_3_(THF)] **5**. Upon addition of 0.5 equivalents of 9-BBN dimer to a solution of crystalline **4**, the ratio of **4** to **5** increases from 61 : 39 to 97 : 3 (Fig. S12‡). A coinciding decrease occurs in the signal at 52.7 ppm and an increase in the signal at 29.4 ppm in the ^11^B NMR spectrum (Fig. S13‡). In contrast, addition of 2 equivalents of DABCO to a solution of **4** results in **5** as the only hydridic species in the ^1^H NMR spectrum (Fig. S14‡), with the only signals in the ^11^B NMR spectrum at 52.7 ppm and the DABCO·9-BBN adduct at 2.38 ppm (Fig. S15‡). A minor unknown species is also formed upon dissolution of **4** in d_8_-THF that displays a sharp singlet at 57.4 ppm in the ^11^B NMR spectrum that diminishes upon addition of either DABCO or 9-BBN.

### Catalytic hydroboration of N-heteroaromatics

**Scheme 5:**
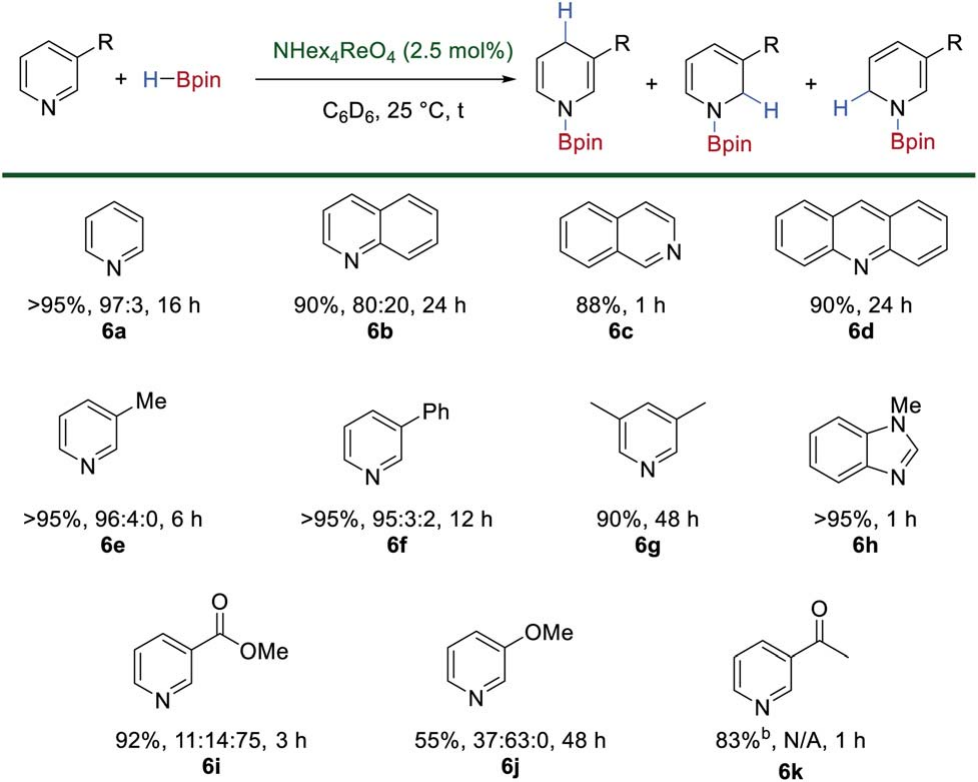
Catalytic hydroboration of N-heteroaromatics using [N(hexyl)_4_][ReO_4_] as a pre-catalyst and HBpin. For **6k** reduction is observed exclusively at the carbonyl when 1 equivalent of HBpin is used.

The simple synthesis of **1** by exhaustive deoxygenation of [ReO_4_]^−^ and the presence of both hydride and boron-based ligands should favor its exploitation in catalytic hydroboration chemistry. Indeed, using [N(hexyl)_4_][ReO_4_] (2.5 mol%) as a bench-stable pre-catalyst with HBpin (1.5 or 2.0 equiv.) results in the catalytic hydroboration of pyridine to give the 1,4-dihydropyridine (1,4-DHP) product in >95% yield after 16 h at room temperature (Scheme 5).^27^ This represents one of the first examples of the hydroboration of N-heteroaromatics using a high oxidation-state, transition metal catalyst.^28^ The hydroboration of benzannulated substrates proceeds in good yield and regioselectivity, with quinoline, isoquinoline and acridine **6b–6d** forming the hydroborated products. 3-Methylpyridine **6e**, 3-phenylpyridine **6f** and 2,6-lutidine **6g** react in good yields and regioselectivities. *N*-Methylbenzimidazole **6h** is hydroborated to the corresponding 1,4-DHP product in high yield. Substrates bearing an electron-withdrawing group such as methyl-3-pyridinecarboxylate **6i** are tolerated in the reaction with no observable reduction of the ester group and relatively short reaction times. 3-Methoxypyridine **6j** is reduced with poor regioselectivity and requires an extended reaction time. Only 1,2-, 1,4- and 1,6-DHP regioisomers are observed in this catalytic system. Interestingly, in the presence of 1 equivalent of HBpin, 3-acetylpyridine **6k** is selectively reduced to the corresponding boroxy ether with no formation of the DHP product observed.

The reactions of *para*- and *ortho*-substituted pyridines are very slow under these conditions with only trace amounts of DHPs observed after extended reaction times. 3-Nitropyridine gives a complex mixture, presumably due to competitive reduction of the nitro group. Similarly, complex mixtures of products are observed for substrates bearing alkynyl, alkenyl and Boc-protected amines. No conversion of iodo-, bromo- and chloro-substituted pyridines to DHPs is seen under these reaction conditions.

The isolated complex **1** is also a competent, albeit sluggish, catalyst for this reaction giving comparable yields and regioselectivity to the catalyst generated *in situ*, a feature which may be due to its reduced solubility. However, when **1** was dissolved in d_5_-pyridine both **1** and **2** are observed in the ^1^H and ^11^B NMR spectra. When monitored over 96 h, the gradual consumption of **1** and **2** is observed along with the formation of multiple new hydride signals in the ^1^H NMR spectrum which eventually resolve into a singlet at *δ* −2.63 ppm (Fig. S6‡). In the ^11^B NMR spectrum a signal at *δ* 23.9 ppm is observed to increase in intensity over time and is indicative of N–Bpin bond formation (Fig. S7‡). These observations suggest that **1** serves as a reservoir for the more reactive, bis(boron) hydride complex **2** through substitution of HBpin by the pyridine substrate. Subsequent boration is likely to proceed by an inner-sphere mechanism of hydride transfer to the activated pyridine substrate, although the 1,4-selectivity seen is unusual for a transition-metal hydride catalyst.^29^

## Conclusions

An operationally simple procedure for the synthesis of the reactive rhenium boron–polyhydride anion **1** from bench-stable and commercially available starting materials has been developed. This is a rare of example of a high oxidation-state, transition metal complex of a boron–hydride ligand. Anion **1** exhibits diverse reactivity including stoichiometric C–H borylation, borane ligand substitution, and the catalytic hydroboration of N-heteroaromatics. The methods described could provide a general route to a range of reactive boron–hydride complexes from other, commercially available, metal oxo anions such as RuO_4_^−^, WO_4_^2−^ and MoO_4_^2−^. These complexes may have wider implications in the catalytic hydroboration of unsaturated organic molecules and in the catalytic C–H borylation of arenes in the presence of stabilising ligands. These aspects are currently under further investigation in our laboratory.

## Conflicts of interest

There are no conflicts to declare.

## Supplementary Material

SC-011-D0SC03458D-s001

SC-011-D0SC03458D-s002
