# Supplementary material for: Synthesis and structures of anionic rhenium polyhydride complexes of boron–hydride ligands and their application in catalysis
Source: Chem Sci. 2020 Sep 9;11(36):9994–9. doi: 10.1039/d0sc03458d (PMC8162066; doi:10.1039/d0sc03458d)
Supplement: SC-011-D0SC03458D-s001 [file SC-011-D0SC03458D-s001.pdf]

Electronic Supplementary Material (ESI) for Chemical Science.
This journal is © The Royal Society of Chemistry 2020

**Supporting information**

# Synthesis and Structures of Anionic Rhenium Polyhydride Complexes of Boron-Hydride Ligands and their Application in Catalysis

Liam J. Donnelly, Simon Parsons, Carole A. Morrison, Stephen P. Thomas* and Jason B. Love*

University of Edinburgh, Joseph Black Building, David Brewster Road, Edinburgh, EH9 3FJ.

**Table of Contents**

1. General Experimental Details 1
2. Synthesis and reactivity of **1** and **4** 3
3. Hydroboration catalysis 5
4. NMR Spectra 12
5. IR Spectra 37
6. Crystallographic Details 38
7. Geometry optimisation of the [$ReH_7(Bpin)_3$] anion 44
8. References 47

## 1.0 General Experimental Details

The syntheses of all air- and moisture-sensitive compounds were undertaken using standard Schlenk techniques. Vacuum Atmospheres and MBraun glove boxes were used to manipulate and store air- and moisture-sensitive compounds under an atmosphere of dried and deoxygenated dinitrogen. All glassware was dried in an oven at 160 °C, allowed to cool under $1 \times 10^{-3}$ mbar vacuum, and then purged with nitrogen. Pyridine was distilled from potassium and stored over 4 Å molecular sieves. HBpin was purchased from Sigma Aldrich

and freeze−pump−thaw degassed and vacuum-transferred prior to use. 18-c-6 was purified by recrystallisation prior to use. All other reagents were purchased from Sigma Aldrich, Alfa Aesar, Acros Organics, Tokyo Chemical Industries UK, Fluorochem, Fisher Scientific UK and Apollo Scientific or synthesised within the laboratory and used without further purification. [N(hexyl)$_4$][ReO$_4$] and [N(butyl)$_4$][ReO$_4$] were prepared by a previously reported method.[1]

THF, toluene, DME, benzene and hexanes were degassed and purified by passage through activated alumina towers and stored over 4 Å molecular sieves. All glassware items, cannulae and Fisher brand 1.2 μm retention glass microfibre filters were dried in a 150 °C oven overnight before use. Deuterated solvents were purchased from Cambridge Isotopes. $C_6D_6$, $d_8$-toluene, $d_8$-THF and $d_5$-pyridine, were boiled over potassium, freeze−pump−thaw degassed and vacuum-transferred prior to use.

$^{1}$H, $^{13}$C{$^{1}$H}, $^{11}$B and $^{29}$Si NMR spectra were recorded on Bruker AVA400, AVA500, or PRO500 spectrometers at 298 K. Chemical shifts are reported in parts per million. All $^{1}$H NMR and $^{13}$C{$^{1}$H} NMR spectra were referenced relative to $SiMe_4$ through a resonance of the employed deuterated solvent or proteo impurity of the solvent; $C_6D_6$ (7.16 ppm), $d_8$-THF (3.58, 1.72 ppm), $d_8$-toluene (7.09, 7.01, 6.97, 2.08 ppm) and $d_5$-pyridine (8.74, 7.58, 7.22 ppm) for $^{1}$H NMR; $C_6D_6$ (128.0 ppm), $d_8$-THF (67.21, 25.31 ppm), $d_8$-toluene (137.48, 128.87, 127.96, 125.13, 20.43 ppm) and $d_5$-pyridine (150.35, 135.91, 123.87 ppm) for $^{13}$C{$^{1}$H} NMR. $^{11}$B and $^{19}$F spectra were referenced using external standards of $BF_3(OEt_2)$ and $CCl_3F$ respectively.

Infra-red (IR) spectra were recorded on a Perkin-Elmer Spectrum One FT-IR, or Shimadzu IRAffinity-1 spectrometer (serial no. A213749). Peaks are reported in $cm^{-1}$ with indicated relative intensities: s (strong, 0–33% T); m (medium, 34–66% T), w (weak, 67–100% T), and br. (broad).

Elemental analyses were determined by Mr. Stephen Boyer at the London Metropolitan University and measured in duplicate.

Single crystal X-ray diffraction data for **1** was collected on a Bruker D8 VENTURE diffractometer fitted with a CCD area detector using MoKα radiation (λ = 0.71073 Å) at 100(2) K. Single crystal X-ray data **4** was collected using an Excalibur Eos diffractometer, fitted with a CCD area detector and using MoKα radiation at 120(2) and 220(2) K respectively. Structures were solved using ShelXT by direct methods or intrinsic phasing and refined using a full-matrix least-squares refinement on $|F|^2$ using ShelXL.[2-3] All programs were used within the Olex suite.[4] All hydrogens atoms were placed geometrically and refined

by using a riding model, with the exception of the hydrides in compound **4** which were located in the difference Fourier map and were isotropically refined with restraints as appropriate. Crystallographic data have been deposited with CCDC (1979241 and 1979242).

## 2.0 Synthesis

### 2.1 Synthesis of [K(DME)(18-c-6)][ReH$_4$(Bpin)(η$^2$-HBpin)( κ$^2$-H$_2$Bpin)] (1)

HBpin (15 mL) was added to KReO$_4$ (1.00 g, 3.5 mmol) and 18-crown-6 (1.86 g, 7 mmol) and stirred at room temperature. After 5 days, the HBpin was evaporated under vacuum (recovered HBpin was recycled for further use) to give a light brown solid. Et$_2$O (20 mL) was added to the crude solid at 0 °C and the mixture was stored at −20 °C for 16 h to precipitate a light brown solid, which was isolated by filtration and dried under vacuum. The solids were dissolved in DME (30 mL) the solution was stored at −20 °C for 16 h to give colourless crystals of **1** which were collected by cannula filtration at 0 °C, washed with cold Et$_2$O (5 mL) and dried under vacuum to give **1** as a colourless solid (1.90 g, 56 %) which was indefinitely stable when stored at −20 °C in a glovebox. Elemental analysis. Calculated for C$_{34}$H$_{77}$B$_3$KO$_{14}$Re (Mr = 968.48 g mol$^{-1}$): C, 42.20; H, 8.02 %. Found: C, 41.56; H, 7.93 %. $^1$H NMR (500 MHz, toluene-d$_8$): δ$_H$ 3.37 (s, 24H), 3.31 (s, 4H, DME), 3.12 (s, 6H, DME), 1.23 (s, 36H), -7.21 (s, 7H). $^{13}$C{$^1$H} NMR (126 MHz, toluene-d$_8$): δ$_C$ 81.7, 72.3, 70.0, 58.6, 25.2. $^{11}$B[$^1$H] NMR (toluene-d$_8$, 300 K): δ $^{11}$B NMR (160 MHz, toluene-d$_8$): δ$_B$ 45.0, 31.3 (Ar-Bpin), 28.6 (d, J = 117.8 Hz, HBpin). IR (ATR): ν 1950 (br.w, Re-H), 1850 (br.w, Re-H). Due to the volatility of the DME ligand bound to potassium the integration for the DME signals in the $^1$H NMR spectrum is low and the elemental analysis is low in C.

### 2.2 Synthesis of [K(DME)(18-c-6)][Re(κ$_2$-H$_2$BBN)$_4$] (4)

Solid 9-BBN dimer (101 mg, 0.412 mmol) was added to a solution of **1** (100 mg, 0.103 mmol) in toluene (3 mL) with stirring at room temperature. After 1 hour, a red precipitate and a light red solution formed. The mixture was filtered and the solids were dried under vacuum after which they were dissolved in DME (1 mL) and hexane was layered on to the solution. After 48 hours, large red crystals formed and these were collected by filtration and dried under vacuum to give the desired product **4** as a red solid (90.6 mg, 82 %). This compound exists in equilibrium with a related Re(BBN)$_3$ **5** species when dissolved in donor solvents such as THF through dissociation of one equivalent of HBBN. Elemental analysis. Calculated

for $C_{48}H_{98}B_4KO_8Re$ (Mr = 1072.68 g mol$^{-1}$): C, 53.79; H, 9.22 %. Found: C, 52.88; H, 9.17 %. $^1$H NMR (500 MHz, THF-d$^8$): $\delta_H$ 3.63 (s, 24H), 3.43 (s, 2.88H, DME), 3.27 (s, 4.12H, DME), 1.99 – 1.77 (m, 26H), 1.66 (m, 8H), 1.56 (m, 10H), 1.46 (m, 8H), 1.43 – 1.36 (m, 6H), -9.02 (s, 4.63H), -9.80 (s, 3.01H). $^{11}$B NMR (160 MHz, THF-d$_8$): $\delta_B$ 57.4 (unknown), 52.7 (**5**), 29.4 (**4**), 13.80 (9-BBN Monomer). IR (ATR): No clear Re-H stretches. Due to the volatility of the DME ligand bound to potassium the integration for the DME signals in the $^1$H NMR spectrum is low and the elemental analysis is low in C.

### 2.3.1 Addition of DABCO to solution of 4

Solid DABCO (4.26 mg, 0.038 mmol) was added to a solution of **4** (20 mg, 0.019 mmol) in THF-d$_8$ in a Youngs' tap NMR tube. The mixture was analysed by NMR spectroscopy and compared with the NMR spectra prior to DABCO addition. Within 10 minutes, **4** was consumed and the single hydride species, **5**, was observed as well as 1 equivalent of DABCO-BBN adduct. $^1$H NMR (500 MHz, THF-d$_8$) δ 3.64 (s, 24H), 3.43 (s, 4H), 3.27 (s, 6H), δ 1.98 – 1.34 (m, 45H, overlapping with DABCO·BBN adduct), -9.80 (s, 7H). $^{11}$B NMR (160 MHz, THF-d$_8$): $\delta_B$ 52.7, 2.4 (d, $J$ = 98.2 Hz, DABCO·BBN).

### 2.4.2 Addition of 9-BBN dimer to solution of 4

Solid 9-BBN dimer (4.6 mg, 0.019 mmol) dimer was added incrementally in two equal portions to a solution of **4** (20 mg, 0.019 mmol) in THF-d$_8$ in a Youngs' tap NMR tube. The reaction was analysed by NMR spectroscopy between additions and compared with the NMR spectra prior to DABCO addition. After the first addition of 0.5 equivalents of 9-BBN dimer, **4** was almost fully consumed and a single hydride species, **4**, was observed as well as free 9-BBN dimer. After the second addition of 9-BBN dimer the ratio of **4**/**5** was 98:2 based on the integration of the hydride signals in the $^1$H NMR spectrum. $^1$H NMR (500 MHz, THF-d$_8$) δ 3.63 (s, 24H), 3.43 (s, 4H), 3.27 (s, 6H), 2.13 – 1.21 (m, 60H, overlap with free 9-BBN), -9.02 (s, 8H). $^{11}$B NMR (160 MHz, THF-d$_8$) $\delta_B$ 57.64 (**5**), 29.29 (**4**), 27.98 (BBN dimer), 13.84 (d, $J$ = 105.0 Hz, BBN monomer).

### 2.5 C-H borylation of toluene using 1

A solution of **1** (100 mg, 0.103 mmol) in toluene (1 mL) was stirred at room temperature for 3 days, during which a dark brown precipitate formed. The solvents was evaporated under vacuum and to the residue was added $C_6D_6$ with trimethoxybenzene (15.7 mg, 0.093 mmol) as an internal standard. NMR analysis of the crude reaction mixture showed that the mono-

borylated product was formed as mixture of *meta* and *para* regioisomers **3a/3b** (41 % NMR yield, 80:20 (*m*/*p*)).

## 3.0 Hydroboration catalysis

### 3.1 Optimisation of hydroboration catalysis conditions[a]

| Entry | Catalyst | Borane | T (°C) | 1,4/1,2[b] | Yield[b] |
|---|---|---|---|---|---|
| 1 | $[N(hexyl)_4][ReO_4]$ | HBpin | 25 | 97:3 | >95% |
| 2 | $[N(butyl)_4][ReO_4]$ | HBpin | 25 | 97:3 | >95% |
| 3 | $ReO_4SiPh_3$ | HBpin | 25 | 71:29 | 28% |
| 4 | $Re_2O_7$ | HBpin | 25 | N/A | <5% |
| 5 | $[N(hexyl)_4][ReO_4]$ | 9-BBN | 25 | 100:00 | 25% |
| 6 | $[N(hexyl)_4][ReO_4]$ | HBcat | 25 | N/A | <5% |
| 7 | $[N(hexyl)_4][ReO_4]$ | $BH_3$ THF | 25 | N/A | <5% |
| 8[c] | $[N(hexyl)_4][ReO_4]$ | HBpin | 80 | 98:2 | >95% |

[a]Reaction conditions: pyridine (0.20 mmol), borane (0.40 mmol, 2 equiv), catalyst (2.5 mol%, 0.005 mmol), mesitylene (internal standard, 10 µL, 0.053 mmol) in 0.4 mL $C_6D_6$ for 16 h.

[b]Regioselectivity and yields were determined by $^1H$ NMR spectroscopy from the crude reaction mixture using mesitylene as an internal standard.

[c]Reaction complete after 6 h.

### 3.2 General procedure for hydroboration catalysis

$[N(hexyl)_4][ReO_4]$ (3.0 mg, 0.005 mmol), the appropriate N-heterocycle **6** (0.20 mmol) and mesitylene (10 µL, 0.053 mmol) as internal standard were combined in $C_6D_6$ (0.4 mL) in an NMR tube sealed with a neoprene rubber stopper. The ratio between the N-heterocycle and mesitylene was measured by $^1H$ NMR spectroscopy. HBpin (58 µL, 0.30 or 0.40 mmol) was added and reaction monitored by $^1H$ NMR spectroscopy to determine the nature and yields of the hydroborated products.

3.2.1 Hydroboration of pyridine (**6a**):

*N*-(4,4,5,5-tetramethyl-1,3,2-dioxaborolan-2-yl)-1,4-dihydropyridine[5]

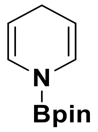

$^{1}H$ NMR (500 MHz, $C_6D_6$): $\delta_H$ 6.52 (dt, *J* = 8.6, 1.7 Hz, 2H), 4.68 – 4.41 (m, 2H), 2.82 (tt, *J* = 3.3, 1.7 Hz, 2H), 0.98 (s, 12H).
$^{11}B$ NMR (160 MHz, $C_6D_6$): $\delta_B$ 23.6.

*N*-(4,4,5,5-tetramethyl-1,3,2-dioxaborolan-2-yl)-1,2-dihydropyridine[5]

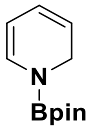


$^{1}H$ NMR (500 MHz, $C_6D_6$): $\delta_H$ 6.79 (dt, *J* = 8.0, 1.9 Hz, 1H), 5.85 – 5.74 (m, 1H), 5.15 – 5.04 (m, 2H), 4.15 (dd, *J* = 4.2, 1.7 Hz, 2H), 1.02 (s, 12H).
$^{11}B$ NMR (160 MHz, $C_6D_6$): $\delta_B$ 23.6.

<u>3.2.2 Hydroboration of quinoline (**6b**):</u>
*N*-(4,4,5,5-tetramethyl-1,3,2-dioxaborolan-2-yl)-1,4-dihydroquinoline[6]

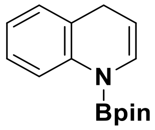


$^{1}H$ NMR (500 MHz, $C_6D_6$): $\delta_H$ 8.11 (dd, *J* = 8.4, 1.2 Hz, 1H), 7.09 (ddd, *J* = 8.8, 7.0, 1.8 Hz, 1H), 6.92 – 6.81 (m, 3H), 4.82 (dt, *J* = 7.8, 3.7 Hz, 1H), 3.32 – 3.30 (m, 2H), 1.00 (s, 12H).
$^{11}B$ NMR (160 MHz, $C_6D_6$): $\delta_B$ 24.6.

*N*-(4,4,5,5-tetramethyl-1,3,2-dioxaborolan-2-yl)-1,2-dihydroquinoline[6]

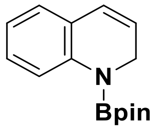


$^{1}H$ NMR (500 MHz, $C_6D_6$): $\delta_H$ 7.77 (d, *J* = 8.2 Hz, 1H), 7.08 (m, 2H), 6.86 (m, 2H), 6.79 (m, 1H), 6.25 (d, *J* = 9.5 Hz, 1H), 5.58 (dt, *J* = 9.6, 4.2 Hz, 1H), 4.14 (dd, *J* = 4.2, 1.8 Hz, 2H), 1.02 (s, 12H).
$^{11}B$ NMR (160 MHz, $C_6D_6$): $\delta_B$ 24.6.

<u>3.2.3 Hydroboration of isoquinoline (**6c**):</u>
1-(4,4,5,5-tetramethyl-1,3,2-dioxaborolan-2-yl)-1,2-dihydroisoquinoline[7]

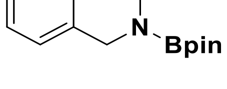

$^{1}H$ NMR (500 MHz, $C_6D_6$): $\delta_H$ 7.02 – 6.97 (m, 1H), 6.89 (dd, *J* = 8.0, 6.8 Hz, 1H), 6.81 (d, *J* = 7.6 Hz, 2H), 6.73 (d, *J* = 7.3 Hz, 1H), 5.62 (d, *J* = 7.5 Hz, 1H), 4.61 (s, 2H), 1.03 (s, 12H).
$^{11}B$ NMR (160 MHz, $C_6D_6$): $\delta_B$ 23.6.

3.2.4 Hydroboration of acridine (**6d**):

1-(4,4,5,5-tetramethyl-1,3,2-dioxaborolan-2-yl)-1,2-dihydroisoquinoline[7]

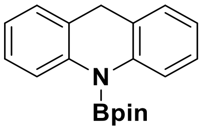


$^{1}H$ NMR (500 MHz, $C_6D_6$): $\delta_H$ 7.81 (d, *J* = 8.4 Hz, 2H), 7.19 – 7.10 (m, 2H), 6.99 – 6.90 (m, 4H), 3.54 (s, 2H), 1.06 (s, 12H).
$^{11}B$ NMR (160 MHz, $C_6D_6$): $\delta_B$ 24.9.

3.2.5 Hydroboration of 3-picoline (**6e**):

3-methyl-*N*-(4,4,5,5-tetramethyl-1,3,2-dioxaborolan-2-yl)-1,4-dihydropyridine[5]

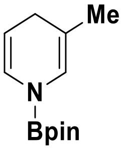


$^{1}H$ NMR (500 MHz, $C_6D_6$): $\delta_H$ 6.59 (dq, *J* = 8.1, 1.6 Hz, 1H), 6.39 (t, *J* = 1.5 Hz, 1H), 4.65 (dt, J = 8.1, 3.3 Hz, 1H), 2.73 (d, *J* = 1.6 Hz, 2H), 1.41 (s, 3H), 1.01 (s, 12H).
$^{11}B$ NMR (160 MHz, $C_6D_6$): $\delta_B$ 23.7.
$^{13}C\{^{1}H\}$ NMR (126 MHz, $C_6D_6$): $\delta_C$ 127.4, 122.2, 110.4, 102.1, 83.3, 28.4, 24.7, 20.8.

3-methyl-*N*-(4,4,5,5-tetramethyl-1,3,2-dioxaborolan-2-yl)-1,2-dihydropyridine[7]

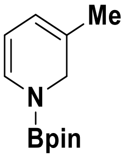


$^{1}H$ NMR (500 MHz, $C_6D_6$): $\delta_H$ 6.62 (d, *J* = 7.8 Hz, 1H), 5.54 – 5.49 (m, 1H), 5.05 (dd, *J* = 7.4, 5.5 Hz, 1H), 4.10 (s, 2H), 1.43 (s, 3H), 1.04 (s, 12H).
$^{11}B$ NMR (160 MHz, $C_6D_6$): $\delta_B$ 23.7.

3.2.6 Hydroboration of 3-phenylpyridine (**6f**)

3-Phenyl-1-(4,4,5,5-tetramethyl-1,3,2-dioxaborolan-2-yl)-1,4-dihydropyridine

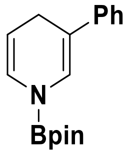

$^1$H NMR (500 MHz, $C_6D_6$): $\delta_H$ 7.28 – 7.23 (m, 2H), 7.19 (d, *J* = 1.2 Hz, 1H), 7.14 – 7.08 (m, 2H), 7.06 – 7.00 (m, 1H), 6.62 (dq, *J* = 8.2, 1.6 Hz, 1H), 4.79 (dt, *J* = 8.1, 3.4 Hz, 1H), 3.17 (dt, J = 3.2, 1.4 Hz, 2H), 1.02 (s, 12H).
$^{11}$B NMR (160 MHz, $C_6D_6$): $\delta_B$ 24.0.

3-Phenyl-1-(4,4,5,5-tetramethyl-1,3,2-dioxaborolan-2-yl)-1,2-dihydropyridine[7]

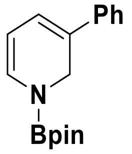

Identified by the characteristic signal, 6.28 (d, *J* = 5.9 Hz, 1H), in the $^1$H NMR spectrum.

3-Phenyl-1-(4,4,5,5-tetramethyl-1,3,2-dioxaborolan-2-yl)-1,6-dihydropyridine

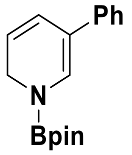

Identified by the characteristic signal, 6.32 (dd, *J* = 9.7, 1.5 Hz, 1H), in the $^1$H NMR spectrum.

3.2.7 Hydroboration of 3,5-lutidine (**6g**):

1-(4,4,5,5-Tetramethyl-1,3,2-dioxaborolan-2-yl)-3,5-dimethyl-1,4-dihydropyridine[5]

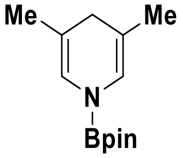

$^1$H NMR (500 MHz, $C_6D_6$): $\delta_H$ 6.45 (s, 2H), 2.59 – 2.52 (s, 2H), 1.48 (s, 6H), 1.04 (s, 12H).
$^{11}$B NMR (160 MHz, $C_6D_6$): $\delta_B$ 23.95.

3.2.8 Hydroboration of 1-methylbenzimidazole (**6h**):

1-(4,4,5,5-tetramethyl-1,3,2-dioxaborolan-2-yl)-3-methyl-2,3-dihydro-1H-benzo[d]imidazole[7]

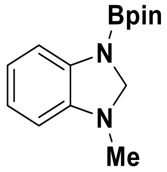

$^{1}H$ NMR (500 MHz, $C_6D_6$): $\delta_H$ 7.48 – 7.41 (m, 1H), 6.85 – 6.73 (m, 2H), 6.30 (dd, *J* = 7.3, 1.3 Hz, 1H), 4.74 (s, 2H), 2.25 (s, 3H), 1.08 (s, 12H).

$^{11}B$ NMR (160 MHz, $C_6D_6$): $\delta_B$ 23.0.

3.2.9 Hydroboration of methyl 3-pyridinecarboxylate (**6i**):

1-(4,4,5,5-tetramethyl-1,3,2-dioxaborolan-2-yl)-1,4-dihydropyridin-3-yl acetate[8]

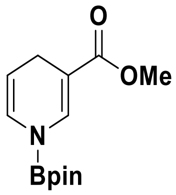

$^{1}H$ NMR (500 MHz, $C_6D_6$): $\delta_H$ 7.78 (s, 1H), 6.31 (d, *J* = 8.0 Hz, 1H), 4.68 (m, 1H), 3.39 (s, 3H), 3.16 (m, 2H), 0.93 ppm (s, 12H).

$^{11}B$ NMR (160 MHz, $C_6D_6$): $\delta_B$ 23.9.

1-(4,4,5,5-tetramethyl-1,3,2-dioxaborolan-2-yl)-1,2-dihydropyridin-3-yl acetate[7]

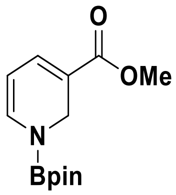

$^{1}H$ NMR (500 MHz, $C_6D_6$): $\delta_H$ 7.09 (dtd, *J* = 6.0, 1.2, 0.8 Hz, 1H), 6.83 (dd, *J* = 7.2, 0.8 Hz, 1H), 4.99 (dd, *J* = 7.2, 6.0 Hz, 1H), 4.59 (d, *J* = 1.2 Hz, 2H), 3.36 (s, 3H), 0.94 (s, 12H).

$^{11}B$ NMR (160 MHz, $C_6D_6$): $\delta_B$ 23.9.

1-(4,4,5,5-tetramethyl-1,3,2-dioxaborolan-2-yl)-1,6-dihydropyridin-3-yl acetate[7]

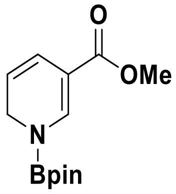

$^1$H NMR (500 MHz, $C_6D_6$): $\delta_H$ 7.97 (t, *J* = 1.2 Hz, 1H), 6.68 (dtd, *J* = 10.0, 2.0, 1.2 Hz, 1H), 4.93 (dtd, *J* = 10.0, 4.0, 1.2 Hz, 1H), 4.01 (dd, *J* = 4.0, 2.0 Hz, 2H), 3.43 (s, 3H), 0.92 (s, 12H).

$^{11}$B NMR (160 MHz, $C_6D_6$): $\delta_B$ 23.9.

3.2.10 Hydroboration of 3-methoxypyridine (**6j**)

3-Methoxy-1-(4,4,5,5-tetramethyl-1,3,2-dioxaborolan-2-yl)-1,4-dihydropyridine[9]

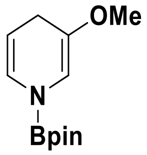


$^1$H NMR (500 MHz, $C_6D_6$): $\delta_H$ 6.57 (dq, I = 8.2, 1.6 Hz, 1H), 6.04 (d, I = 1.1 Hz, 1H), 4.62 (dt, I = 8.0, 3.4 Hz, 1H), 3.18 (s, 3H), 3.07 (dt, I = 3.2, 1.4 Hz, 2H), 1.02 (s, 12H).

$^{11}$B NMR (160 MHz, $C_6D_6$): $\delta_B$ 24.0.

3-Methoxy-1-(4,4,5,5-tetramethyl-1,3,2-dioxaborolan-2-yl)-1,2-dihydropyridine[7]

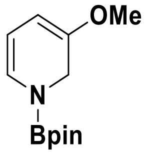


$^1$H NMR (500 MHz, $C_6D_6$): $\delta_H$ 6.48 (d, *J* = 7.2 Hz, 1H), 5.11 (dd, *J* = 7.2, 6.2 Hz, 1H), 4.75 (d, J = 6.1 Hz, 1H), 4.30 (d, *J* = 1.1 Hz, 2H), 3.15 (s, 3H), 1.01 (s, 12H).

$^{11}$B NMR (160 MHz, $C_6D_6$): $\delta_B$ 24.0.

3.2.11 Hydroboration of methyl-3-pyridylketone (**6k**)

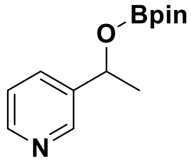


$^1$H NMR (400 MHz, $C_6D_6$): $\delta_H$ 8.71 (d, *J* = 2.3 Hz, 1H), 8.44 (dd, *J* = 4.7, 1.7 Hz, 1H), 7.42 (dt, *J* = 7.9, 2.1 Hz, 1H), 6.74 (ddd, *J* = 7.9, 4.8, 0.9 Hz, 1H), 5.25 (q, *J* = 6.5 Hz, 1H), 1.30 (d, *J* = 6.5 Hz, 3H), 1.00 (s, 6H), 0.97 (s, 6H).

$^{13}$C{$^1$H} NMR (101 MHz, $C_6D_6$): $\delta_C$ 149.2, 148.2, 132.6, 127.4, 123.3, 82.7, 70.8, 25.1, 24.6, 24.5.

$^{11}$B NMR (128 MHz, $C_6D_6$): $\delta_B$ 22.5.

### 3.3 Pyridine hydroboration catalysed by 1

**1** (4.84 mg, 0.005 mmol), pyridine (0.20 mmol) and mesitylene (10 μL, 0.053 mmol) as internal standard were combined in $C_6D_6$ (0.4 mL) in an oven-dried NMR tube sealed with a neoprene rubber stopper. The ratio between the N-heterocycle and mesitylene was measured by $^1H$ NMR spectroscopy. HBpin (58 μL, 0.40 mmol) was added and reaction was monitored by $^1H$ NMR spectroscopy for 80 hours at room temperature. NMR analysis of the crude reaction mixture showed that a mixture of the 1,4 and 1,2 products was formed (78% NMR yield, 95:5 (1,4/1,2).

### 3.4 'Bench-top' pyridine Hydroboration (in air, using 'wet' solvents and reagents)

$[N(hexyl)_4][ReO_4]$ (3.0 mg, 0.005 mmol), pyridine (0.20 mmol) and mesitylene (10 μL, 0.053 mmol) as internal standard were mixed in $C_6D_6$ (0.4 mL) in an oven-dried NMR tube sealed with a neoprene rubber stopper. The ratio between the N-heterocycle and mesitylene was measured by $^1H$ NMR spectroscopy. HBpin (87 μL, 0.60 mmol) was added and reaction was monitored by $^1H$ NMR spectroscopy for 16 hours at room temperature. NMR analysis of the crude reaction mixture showed that a mixture of the 1,4 and 1,2 products was formed (>95% NMR yield, 98:2 (1,4/1,2).

## 4.0 NMR Spectra

[K(DME)(18-c-6)][$ReH_7(Bpin)_3$] **(1)**

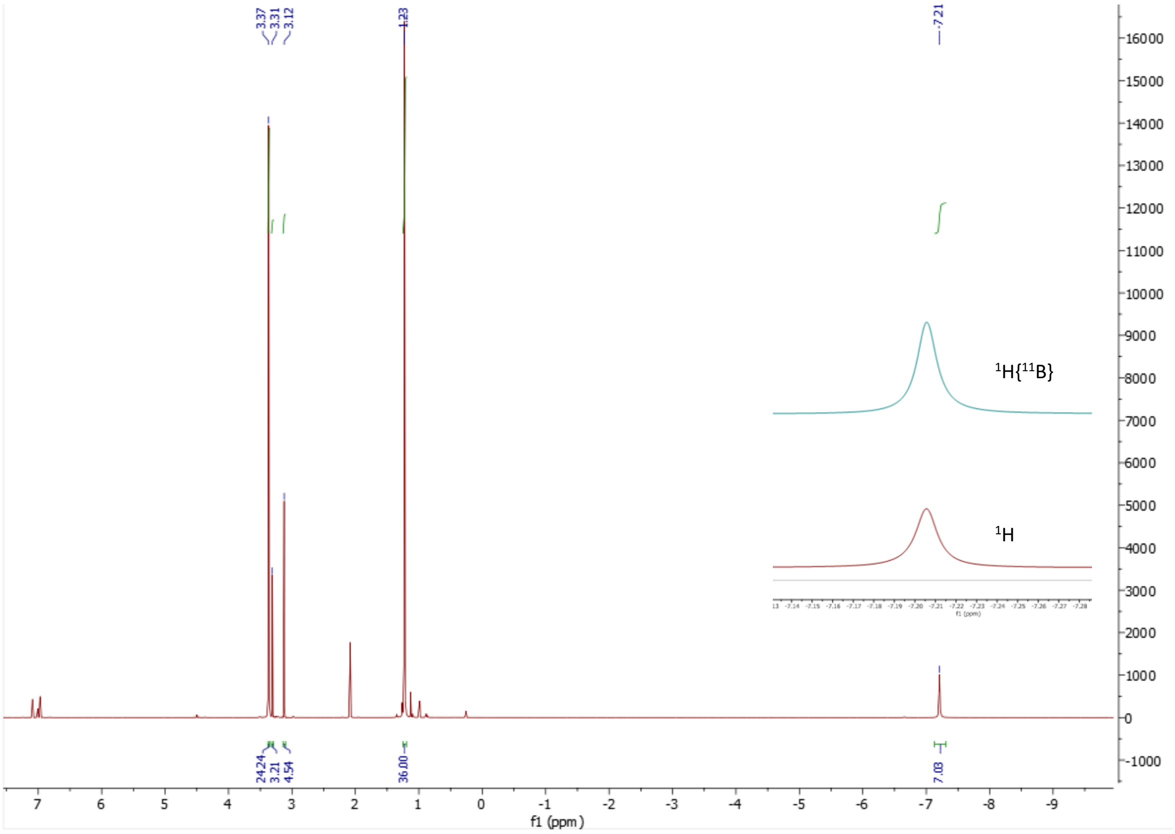


**Figure S1. $^1$H NMR spectrum of 1 in Tol-$d_8$. Insert shows hydride signals upon $^{11}$B decoupling.**

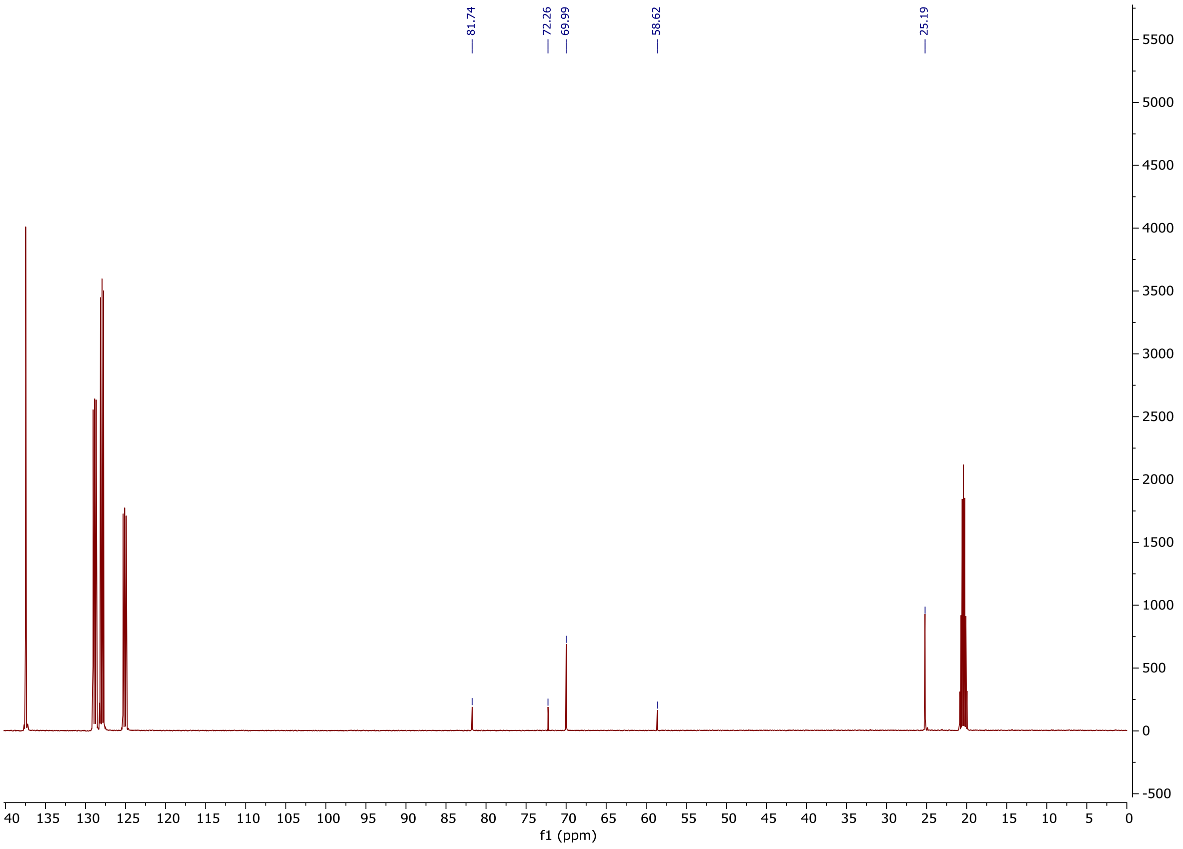


**Figure S2. $^{13}$C NMR spectrum of 1 in Tol-d$_8$.**

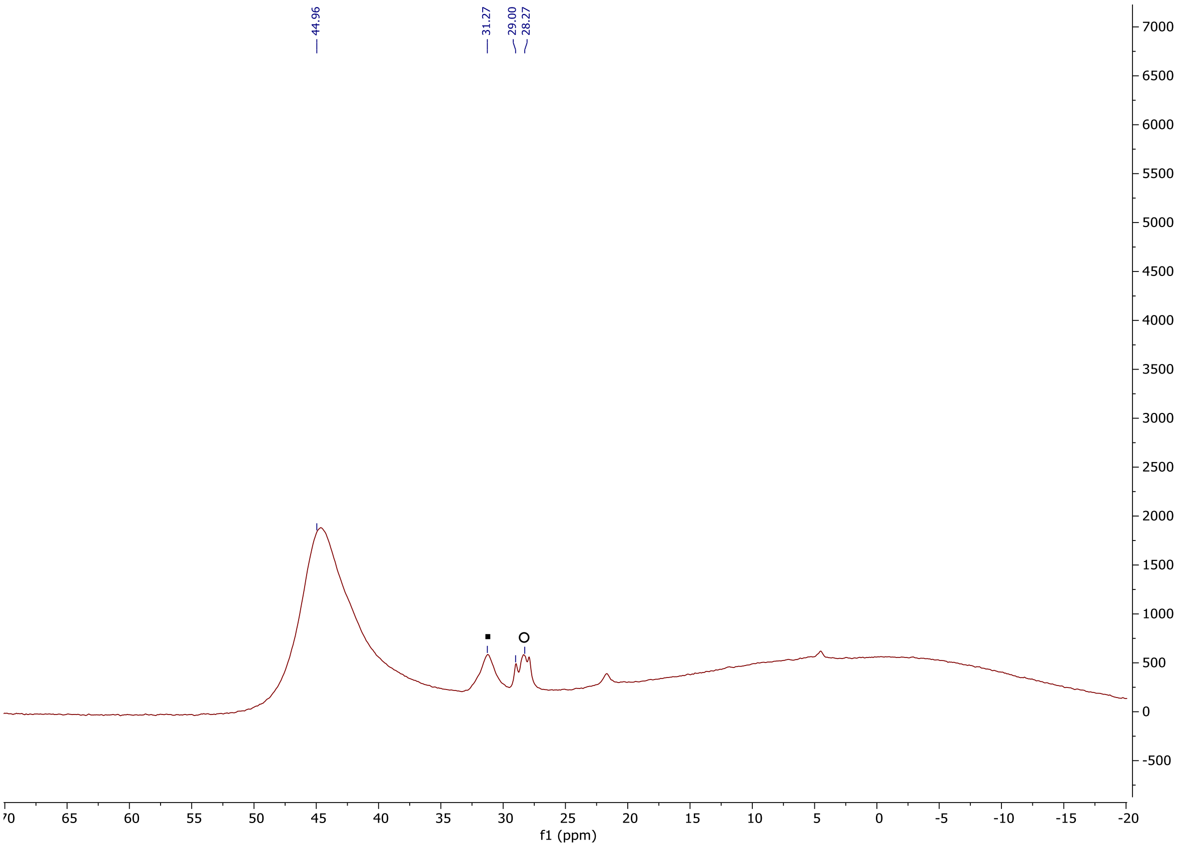


**Figure S3. $^{11}$B NMR spectrum of 1 in Tol-d$_8$. Assignment:** ▪ TolBpin, ○ HBpin.

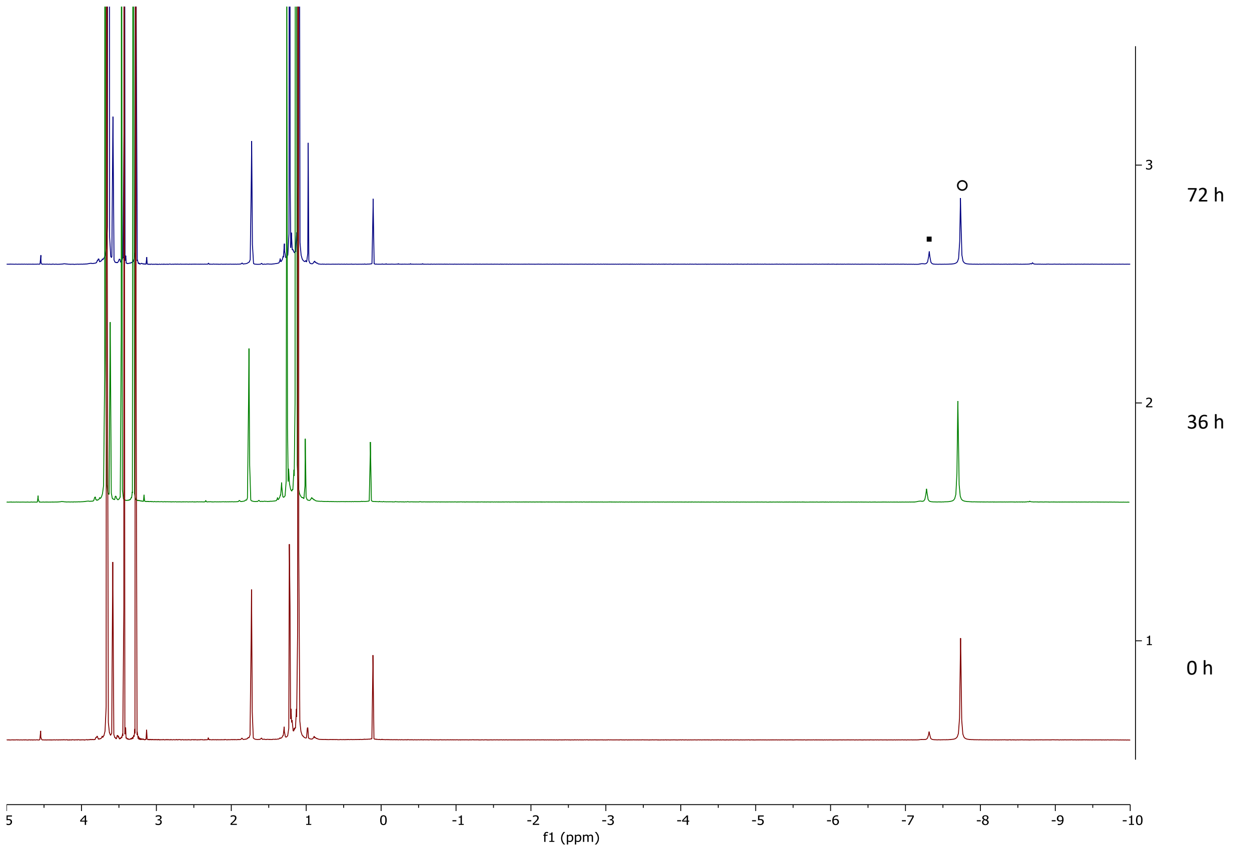


**Figure S4. $^{1}$H NMR spectrum of 1 in THF-d$_8$. Assignment: ▪ 2, ○ 1.**

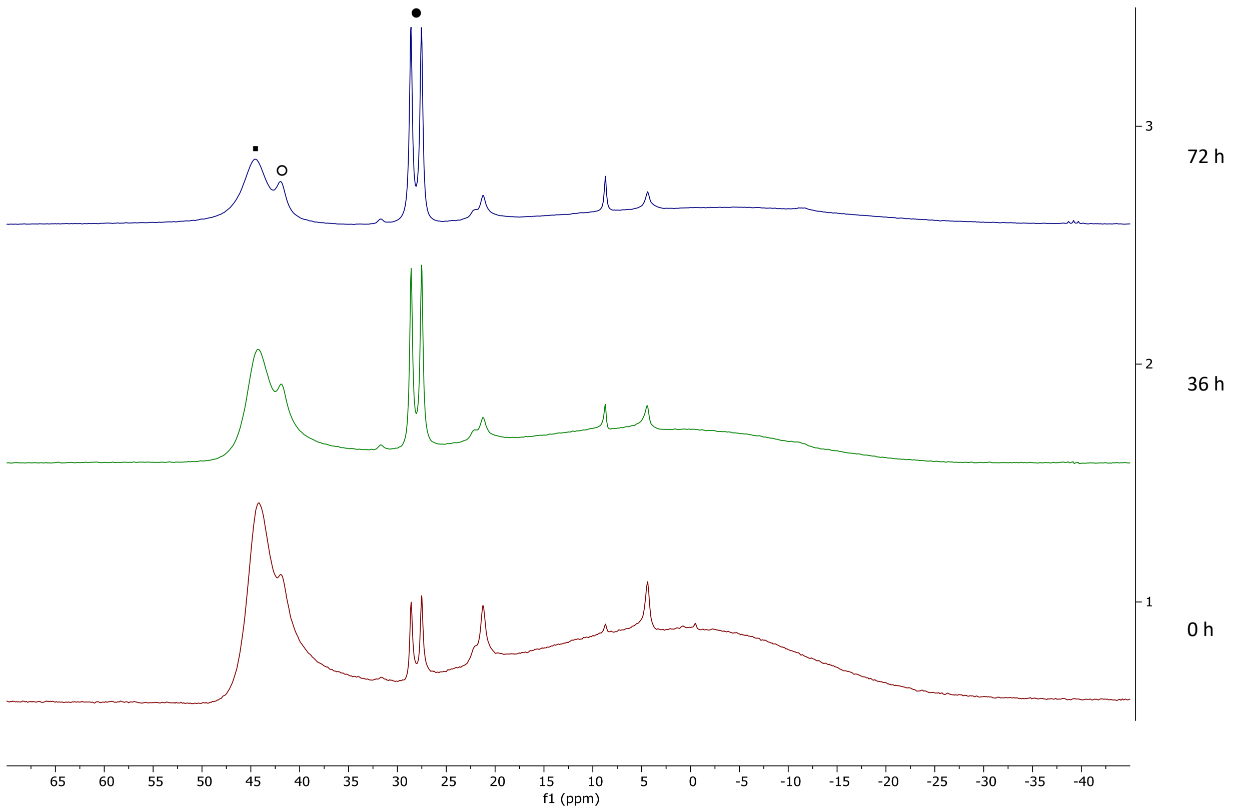


**Figure S5. $^{11}$B NMR spectrum of 1 in THF-d$_8$. Assignment: ▪ 1, ○ 2, ●** HBpin

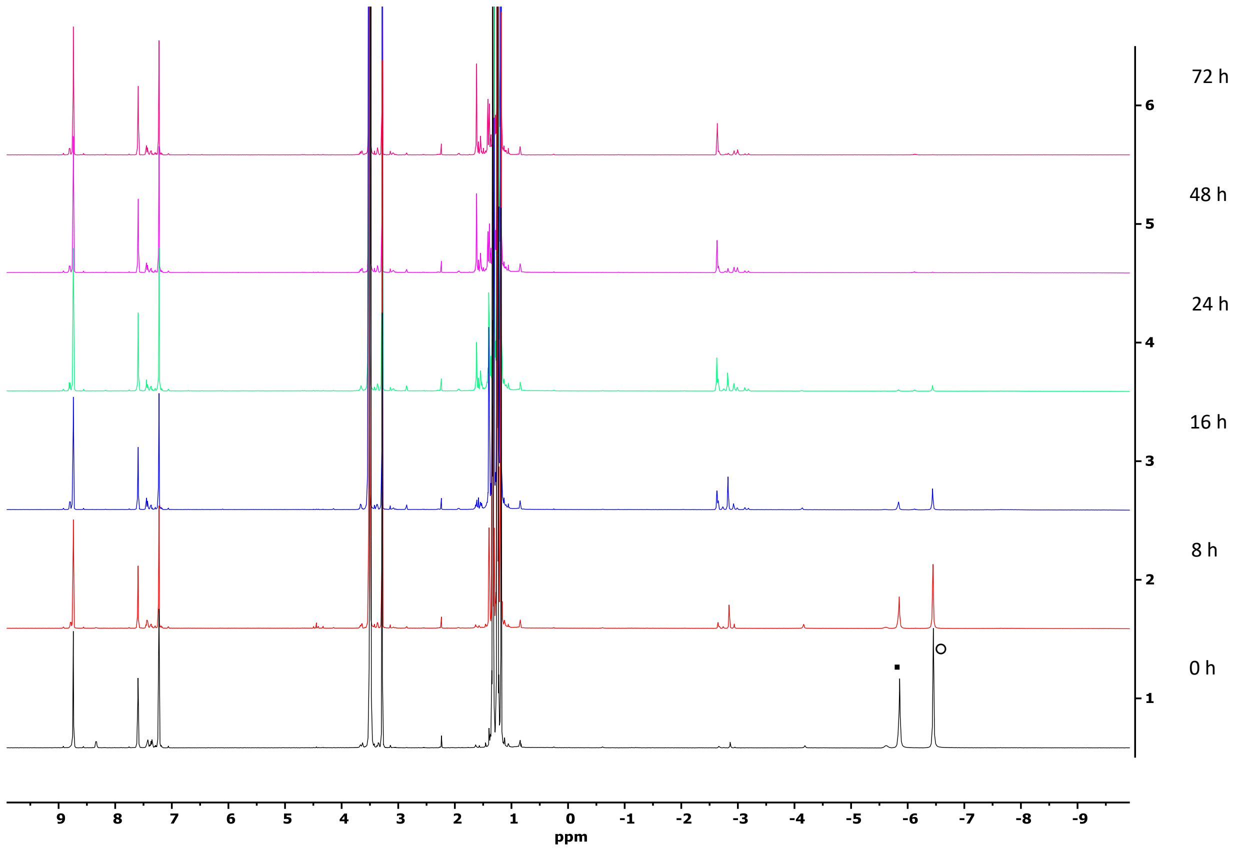


**Figure S6. $^{1}$H NMR spectrum of 1 in pyridine-d5. Assignment: ▪ 1, ○ 2**

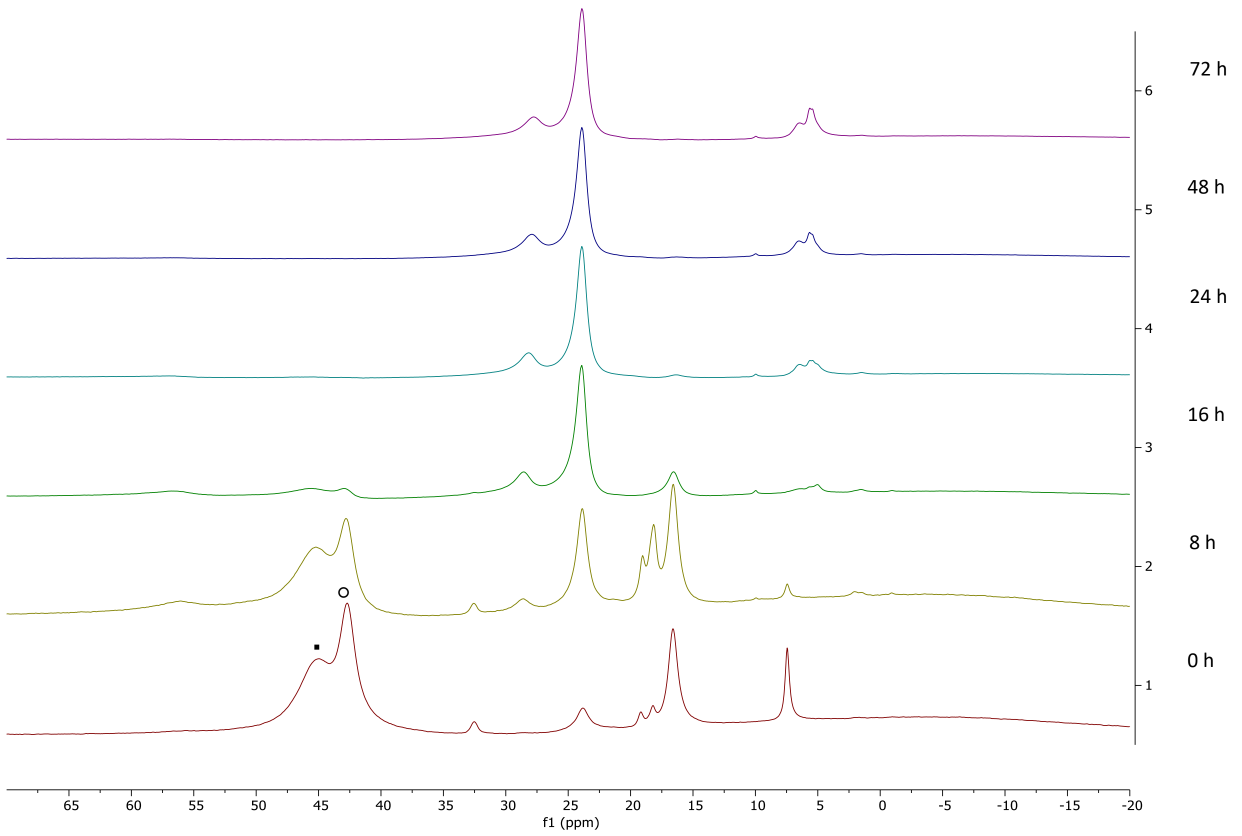


**Figure S7. $^{11}$B NMR spectrum of 1 in pyridine-d5. Assignment: ▪ 1, ○ 2**

## Reaction of $[NBu_4][ReO_4]$ with HBpin

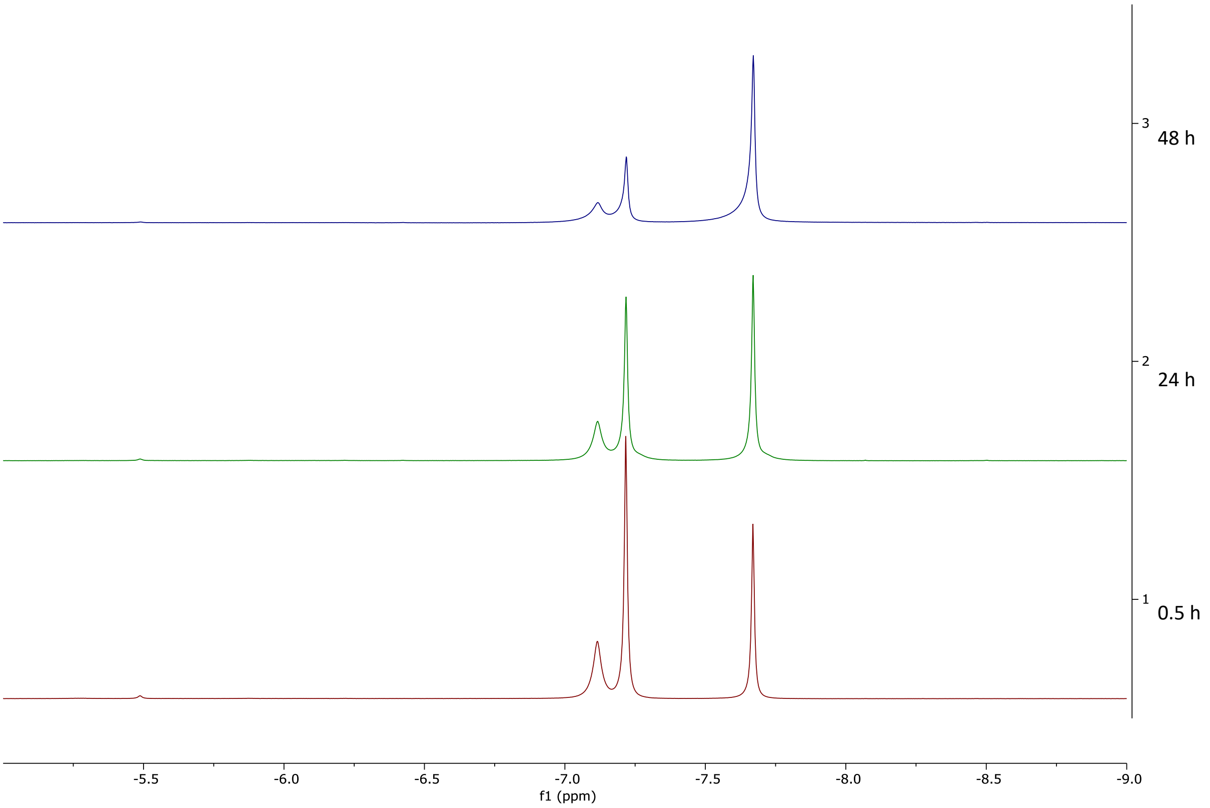


**Figure S8. HBpin (70.7 µL, 0.487 mmol) was added to a solution of $[NBu_4][ReO_4]$ (20 mg, 0.041 mmol) in THF-$d_8$ in a Youngs' tap NMR tube. The mixture was analysed by $^1H$ NMR spectroscopy in the hydride region over the course of 48 hours at room temperature.**

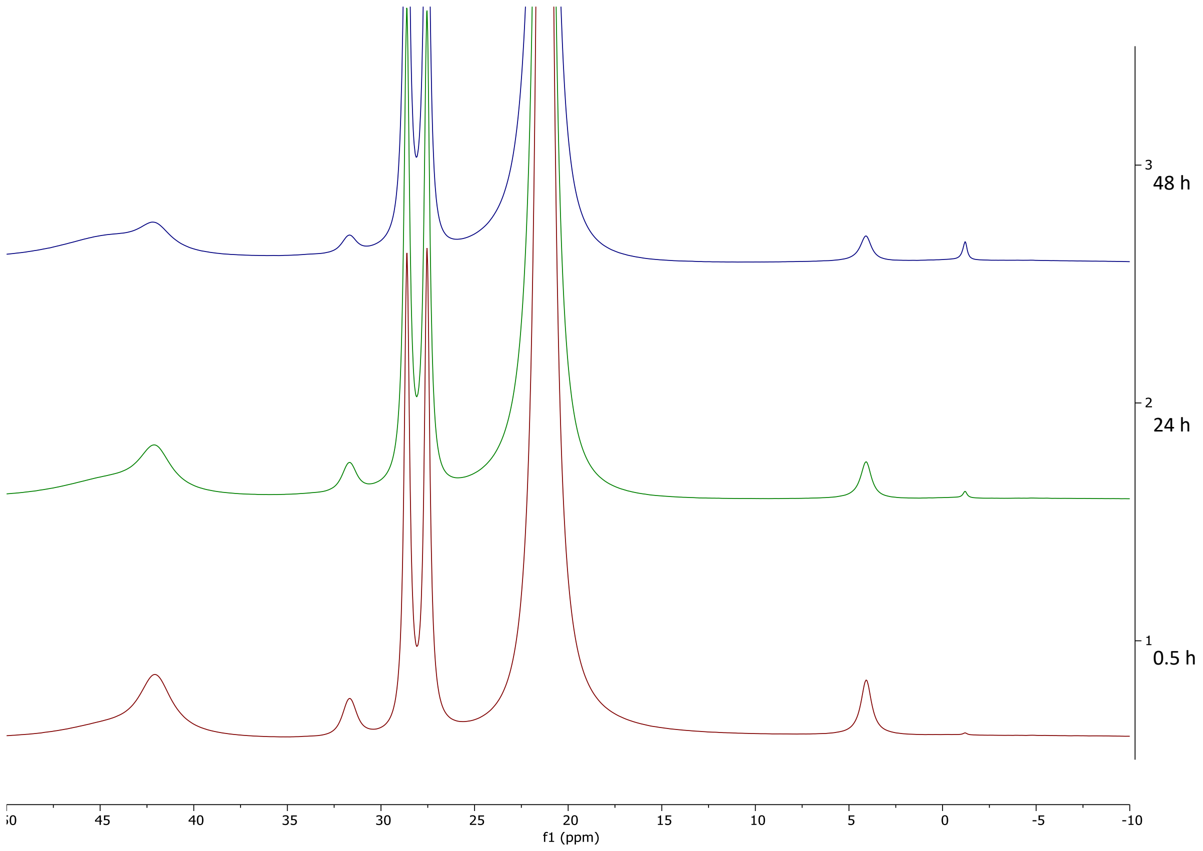


**Figure S9. HBpin (70.7 µL, 0.487 mmol) was added to a solution of [$NBu_4$][$ReO_4$] (20 mg, 0.041 mmol) in THF-$d_8$ in a Youngs' tap NMR tube. The mixture was analysed by $^{11}$B NMR spectroscopy over the course of 48 hours at room temperature.**

## [K(DME)(18-c-6)][Re(κ$_2$-H$_2$BBN)$_4$] **(4)**

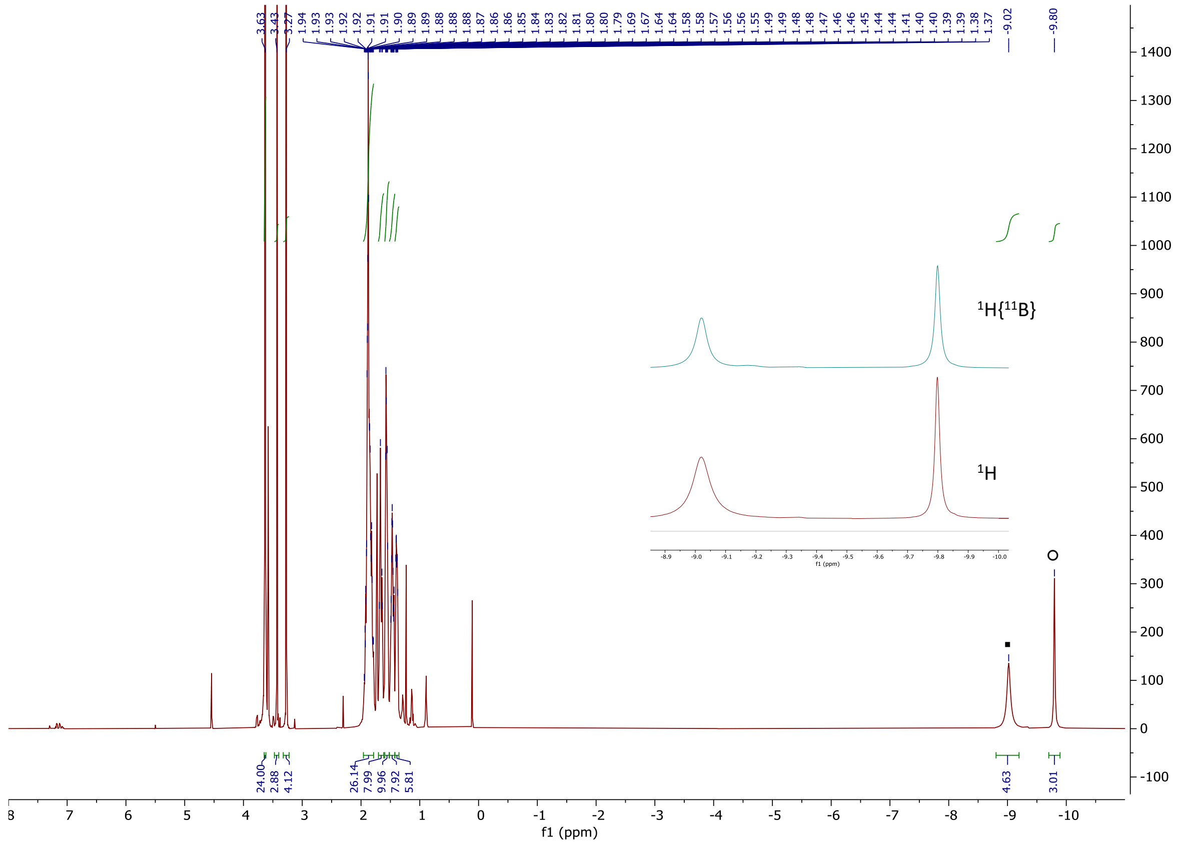


**Figure S10. $^1$H NMR spectrum of 4 in THF-d$_8$. Insert shows hydride signals upon $^{11}$B decoupling. ▪ 4, ○ 5**

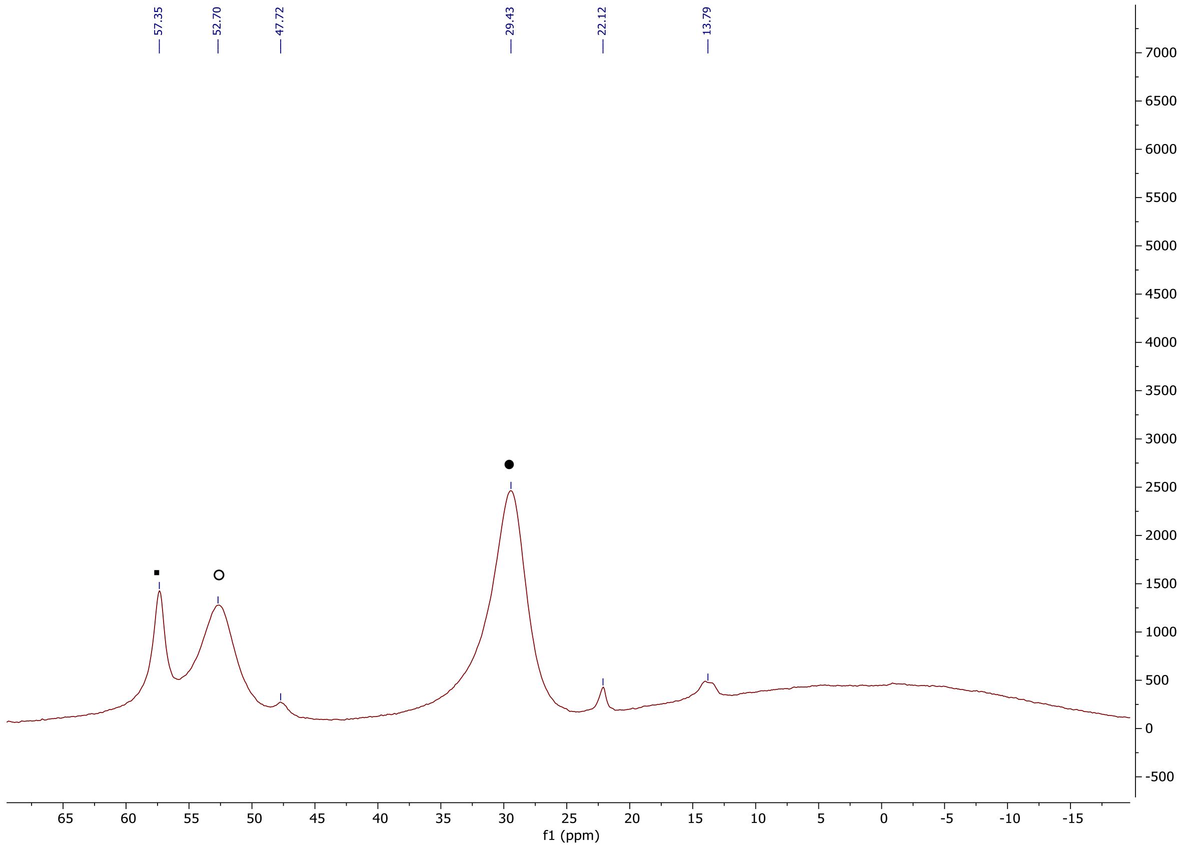


**Figure S11. $^{11}$B NMR spectrum of 4 in THF-d$_8$.** ▪ Unknown, ○ **4**, ● **5**.

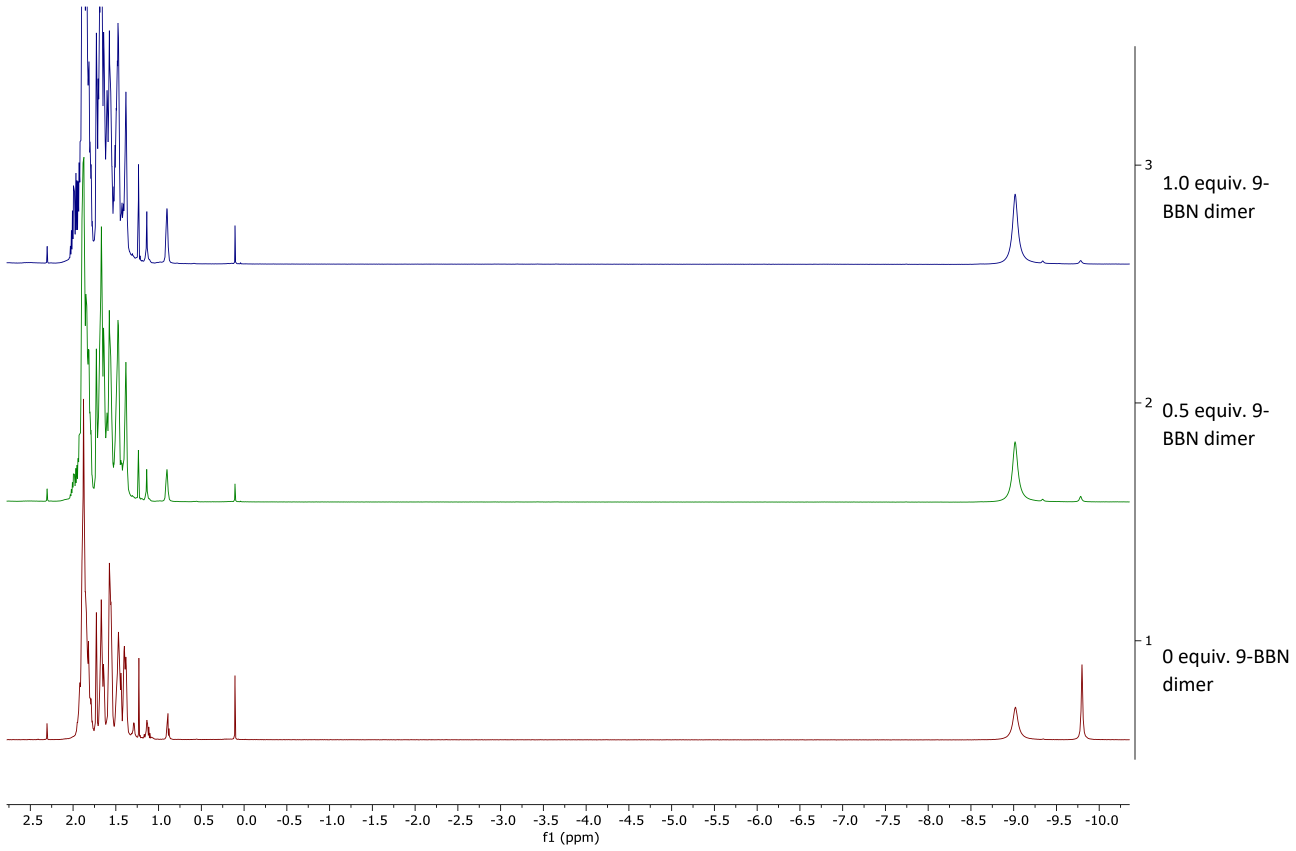


**Figure S12.** $^{1}$**H NMR spectrum of addition of 9-BBN dimer to 4 in THF-d**$_{8}$**.**

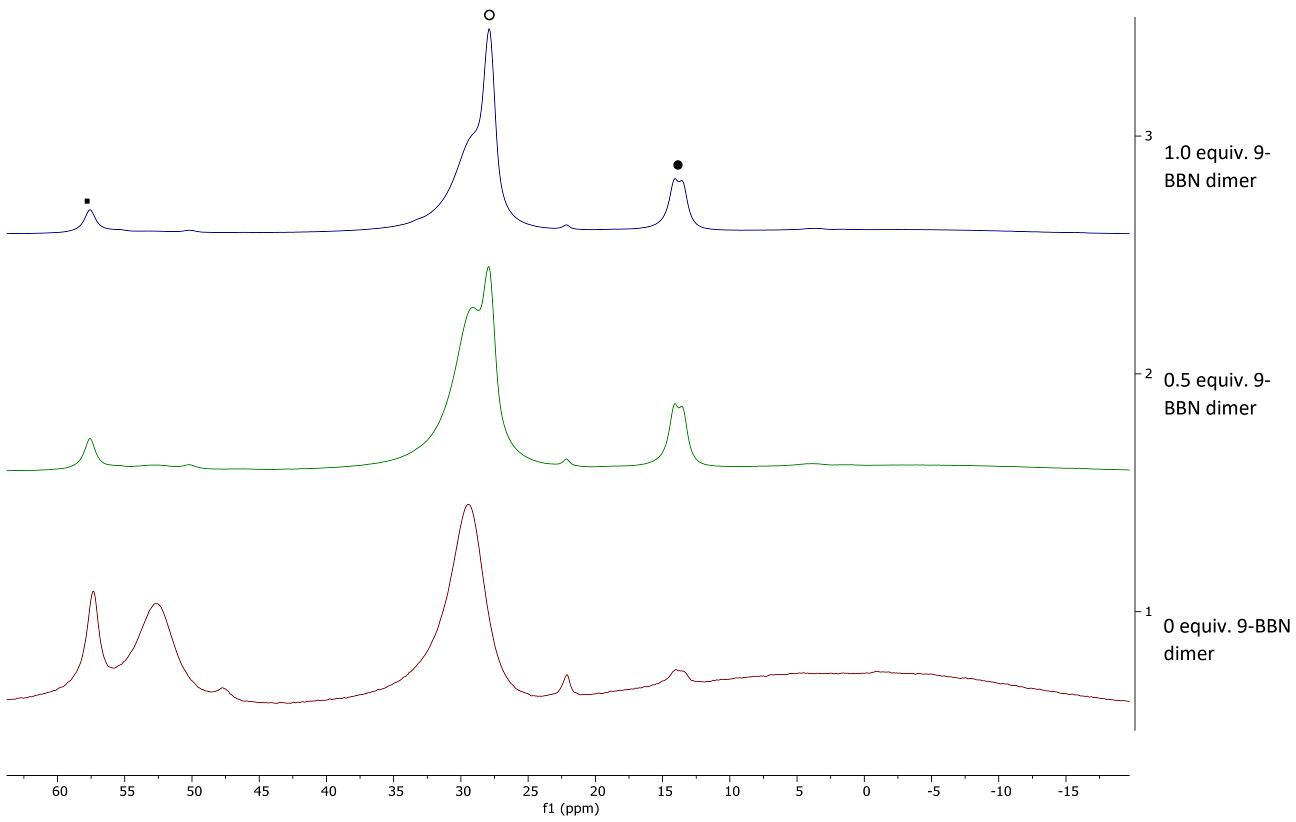


**Figure S13.** $^{11}$**B NMR spectrum of addition of 9-BBN dimer to 4 in THF-d**$_{8}$**.** ▪ Unknown, ○ 9-BBN dimer, ● 9-BBN monomer.

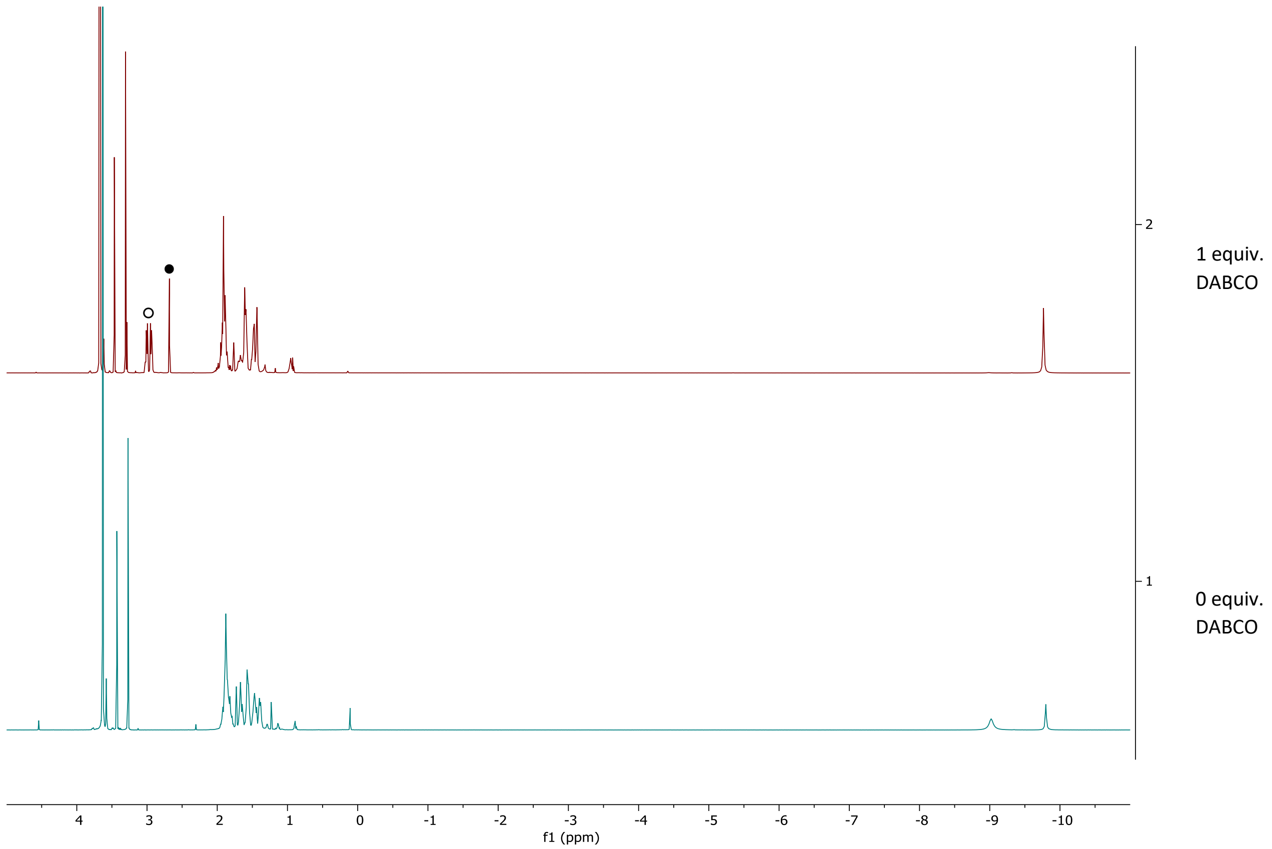


**Figure S14. ¹H NMR spectrum of addition of DABCO to 4 in THF-d$_8$.** ○ DABCO 9-BBN adduct, ● Free DABCO.

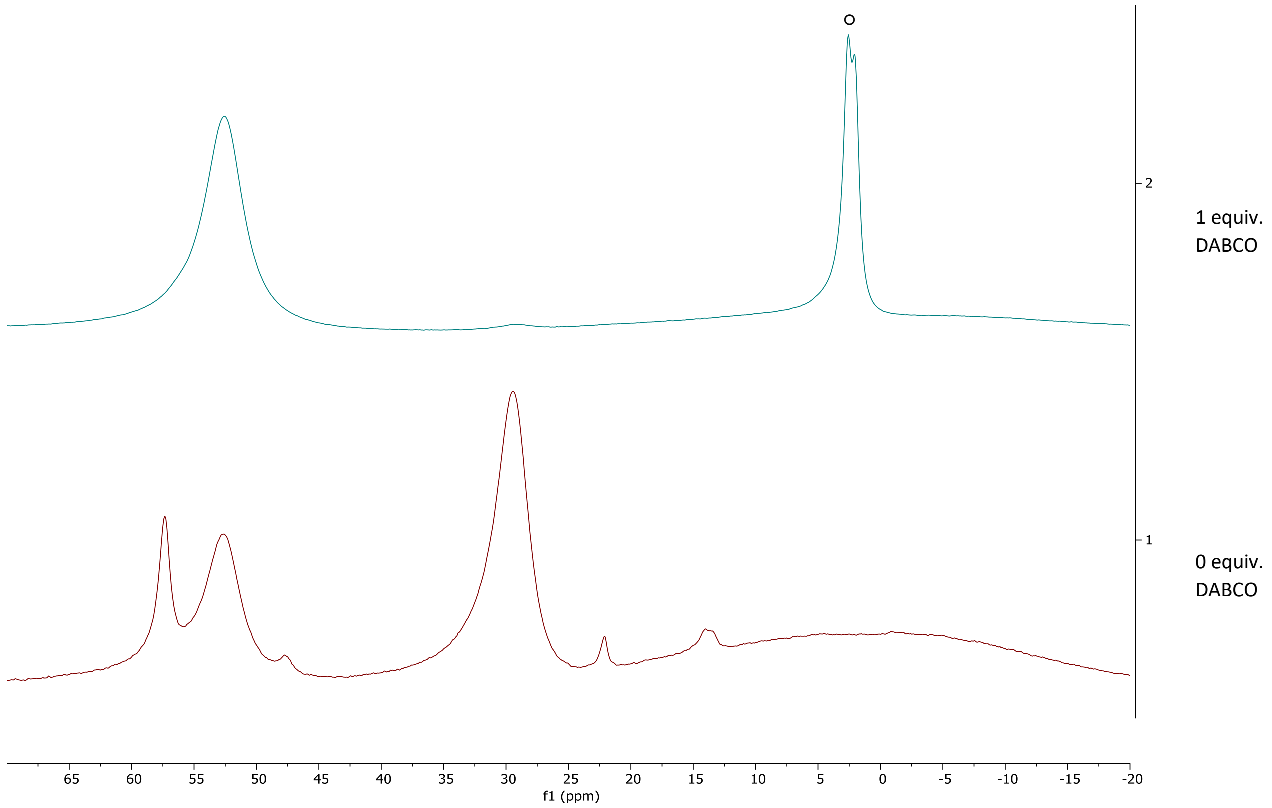


**Figure S15. ¹H NMR spectrum of addition of DABCO to 4 in THF-d$_8$.** ○ DABCO 9-BBN adduct

## C-H borylation of toluene using **(1)**

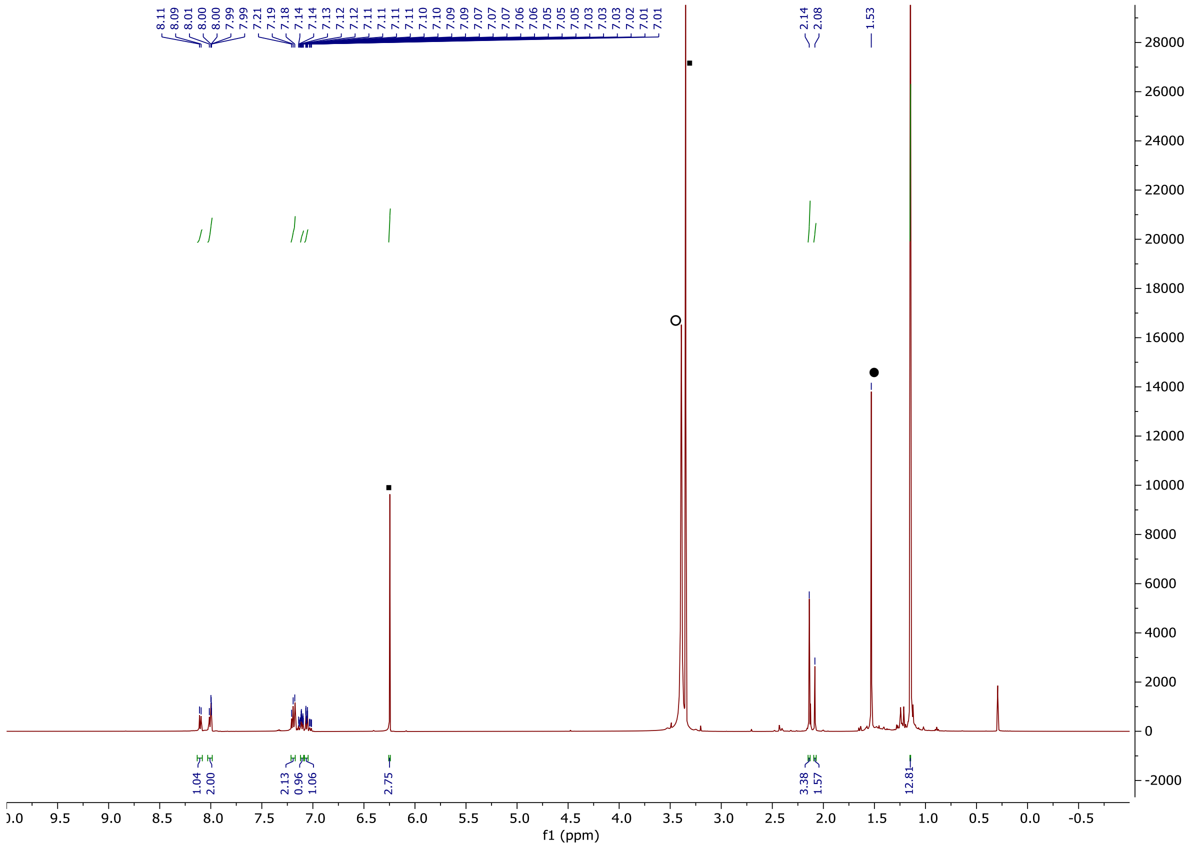


**Figure S16. $^{1}$H NMR spectrum of the C-H borylation of toluene using 1 in $C_6D_6$.** ▪ Trimethoxybenzene internal standard, ○ 18-c-6 **1**, ● Unknown degradation product.

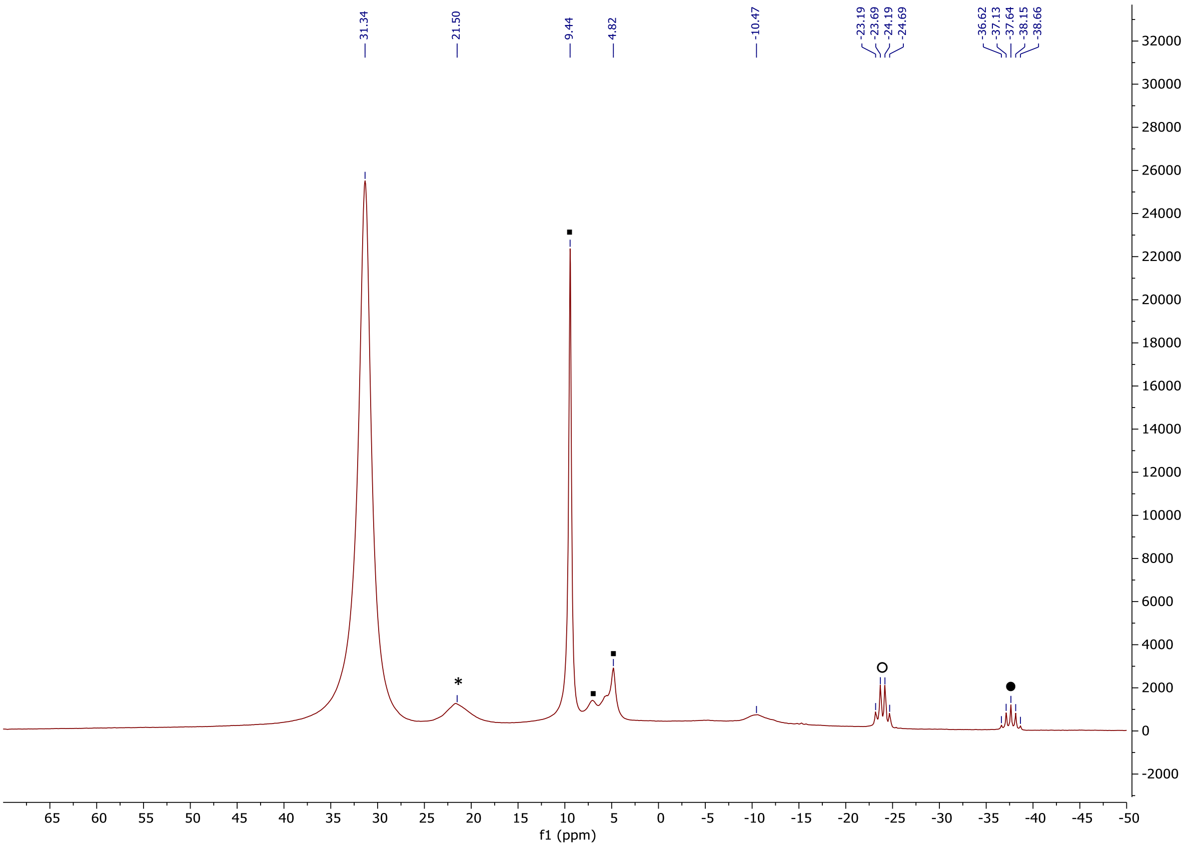


**Figure S17. $^{11}$B NMR spectrum of the C-H borylation of toluene using 1 in $C_6D_6$.** * $O(Bpin)_2/B_2(pin)_3$, ▪ Unidentified degradation products, ○ $BH_3$, ● $BH_4^-$

Hydroboration of pyridine (**6a**):

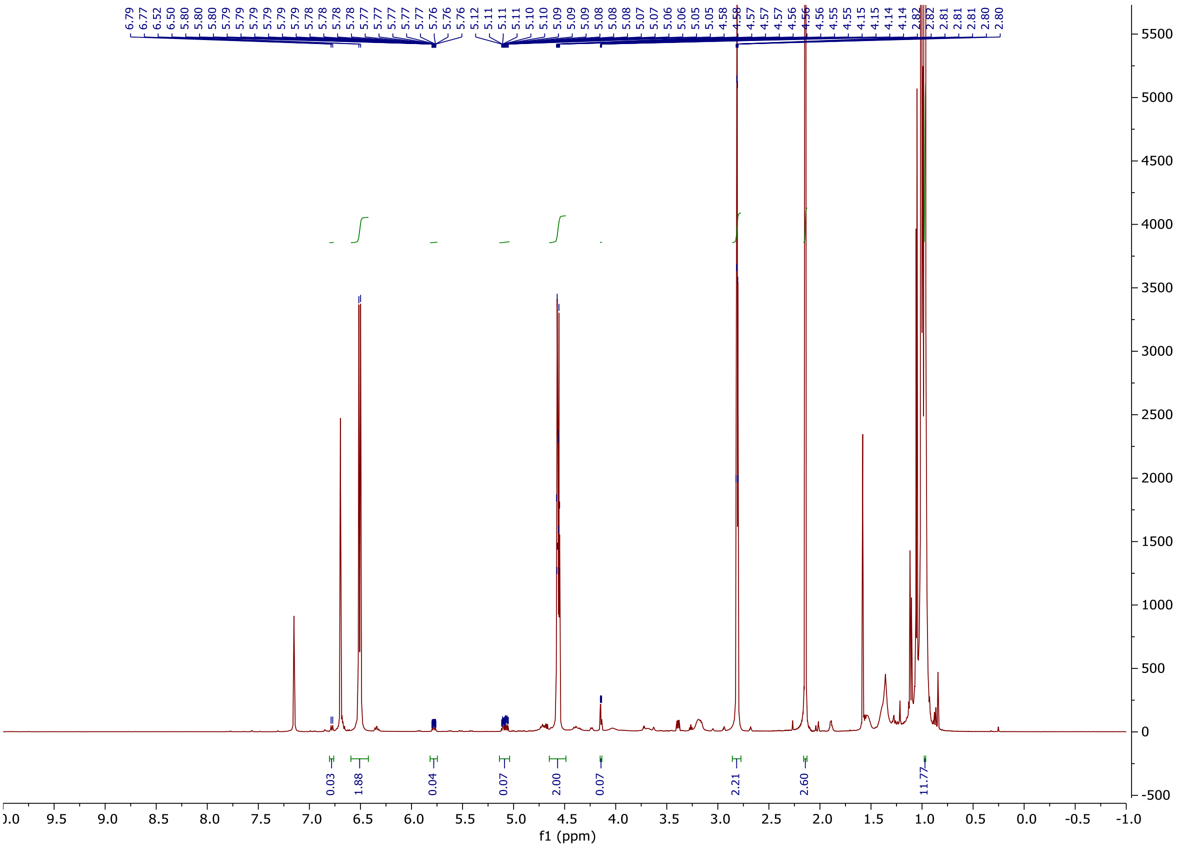

**Figure S18. $^{1}$H NMR spectrum of Hydroboration of 6a in $C_6D_6$.**

Hydroboration of quinoline (**6b**):

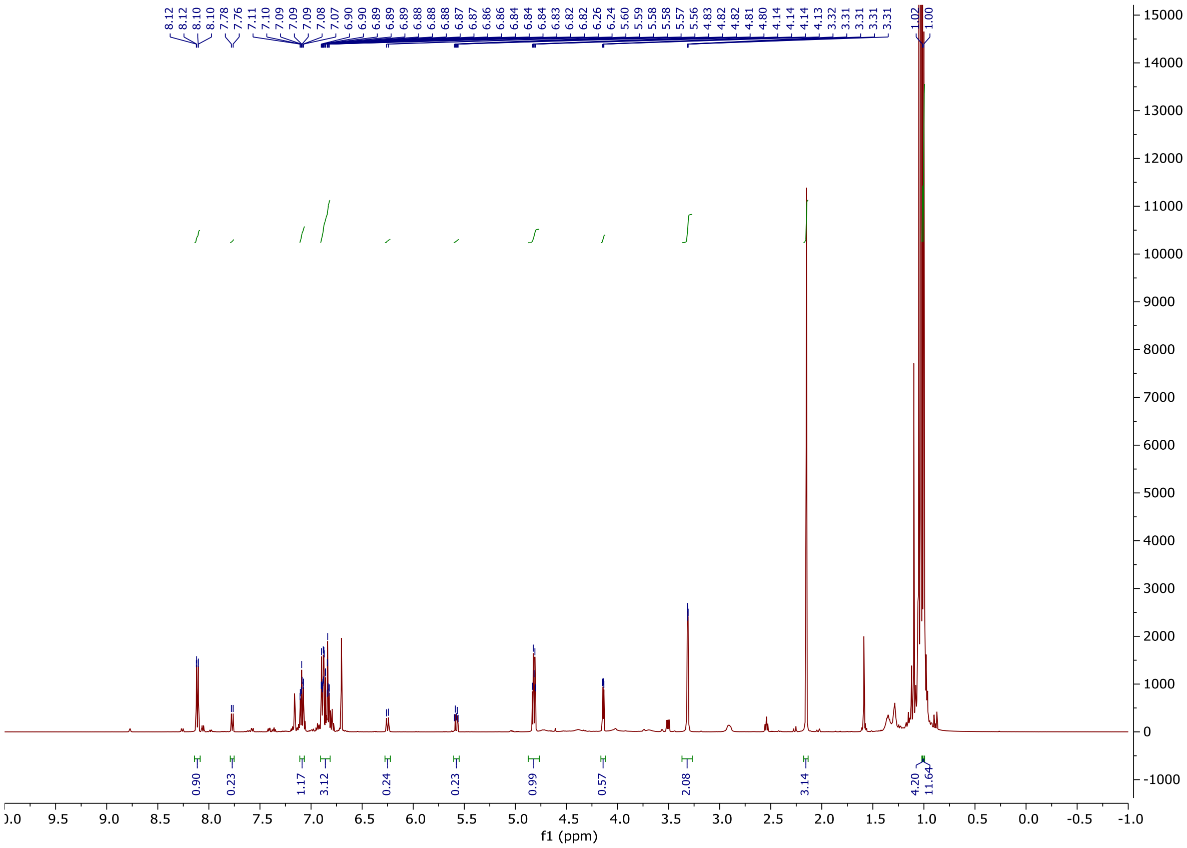

**Figure S19. $^{1}$H NMR spectrum of Hydroboration of 6b in $C_6D_6$.**

Hydroboration of isoquinoline (**6c**):

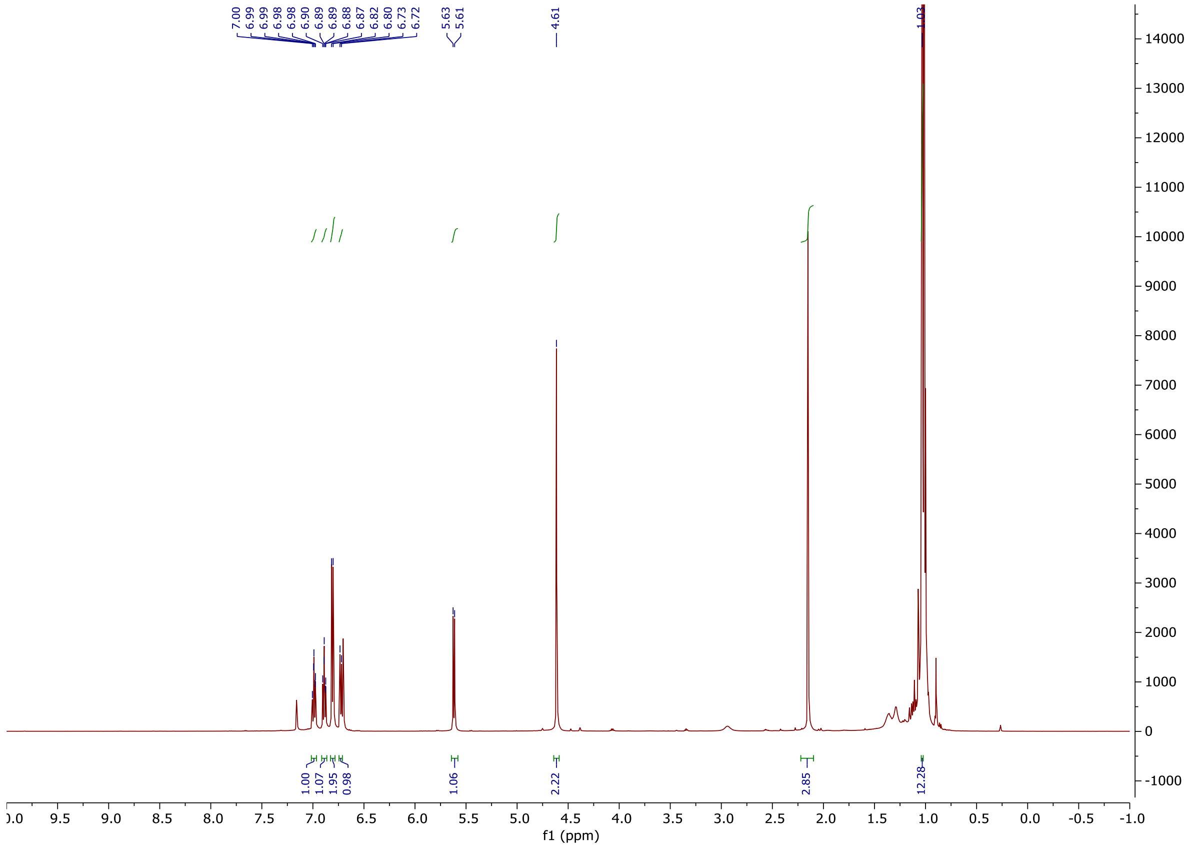


**Figure S20. $^{1}$H NMR spectrum of Hydroboration of 6c in $C_6D_6$.**

Hydroboration of acridine (**6d**):

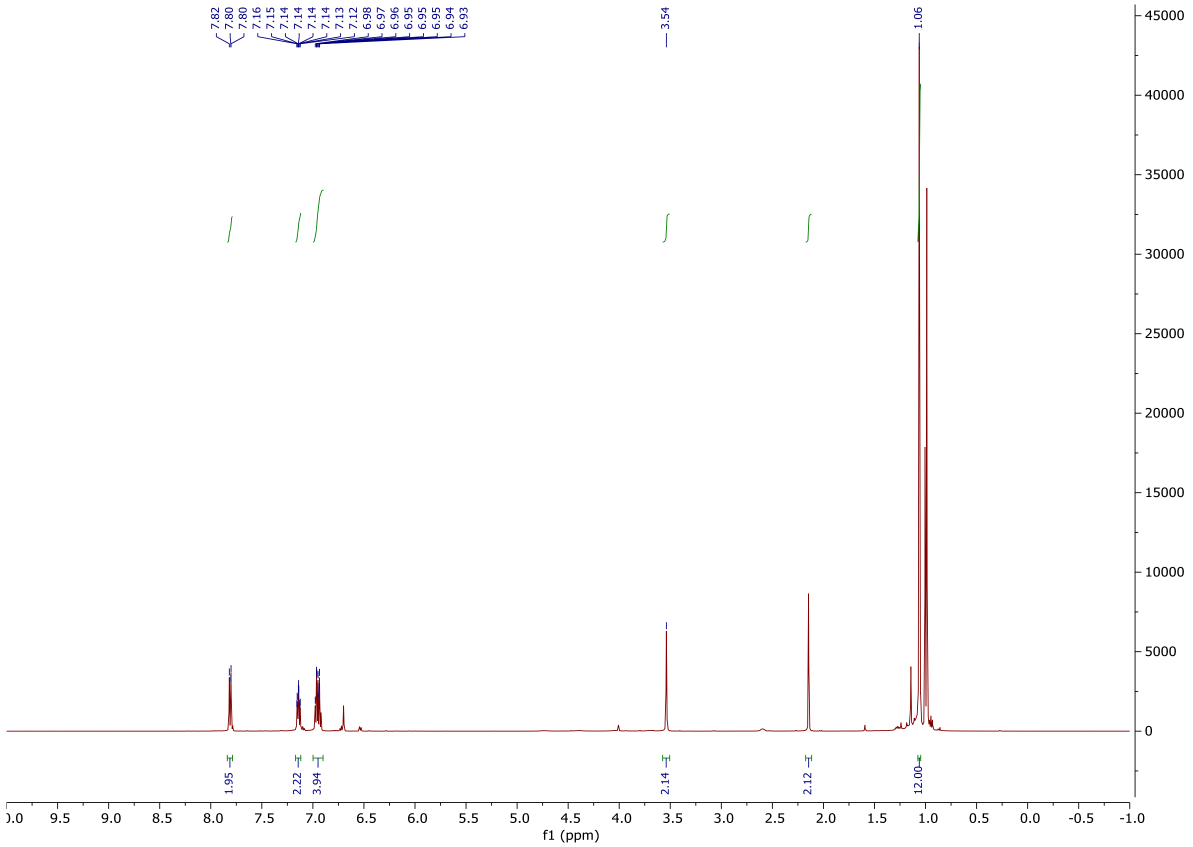


**Figure S21. $^{1}$H NMR spectrum of Hydroboration of 6d in $C_6D_6$.**

Hydroboration of 3-picoline (**6e**):

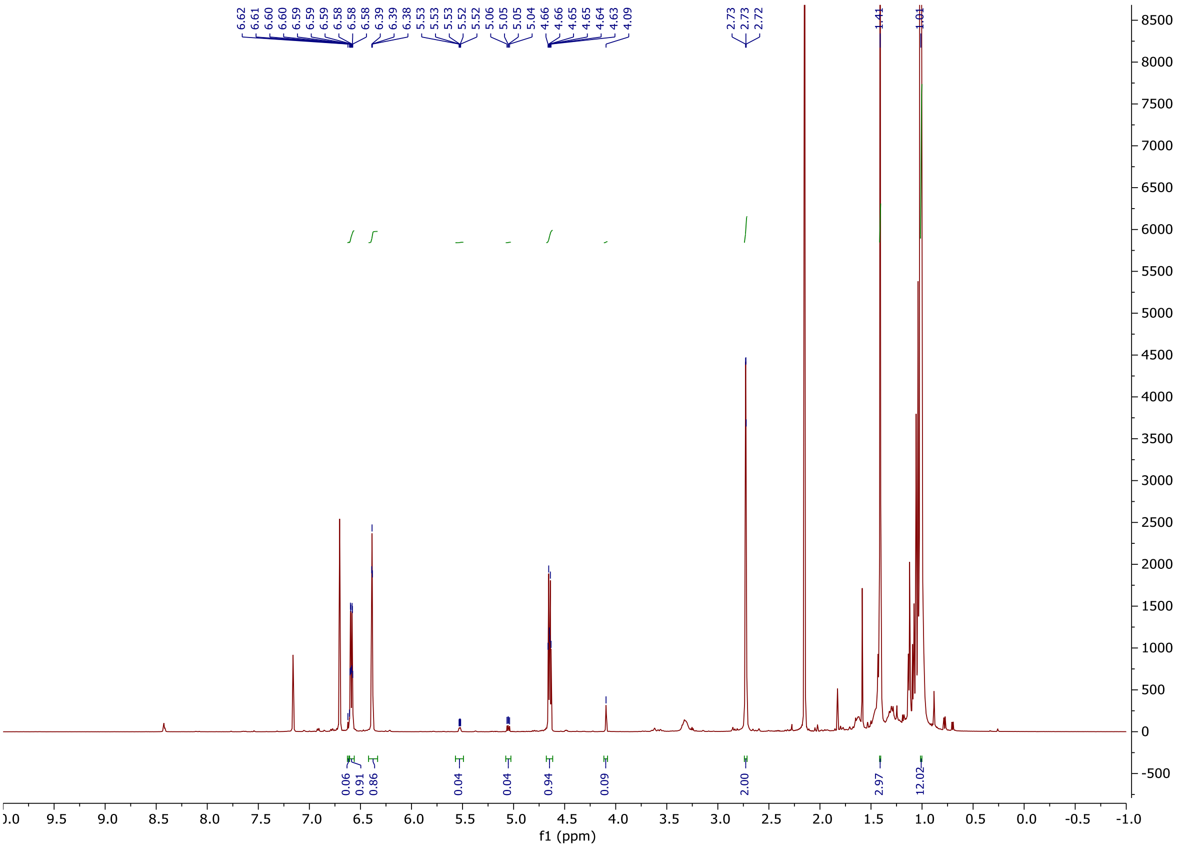


**Figure S22. $^{1}$H NMR spectrum of Hydroboration of 6e in $C_6D_6$.**

Hydroboration of 3-phenylpyridine (**6f**)

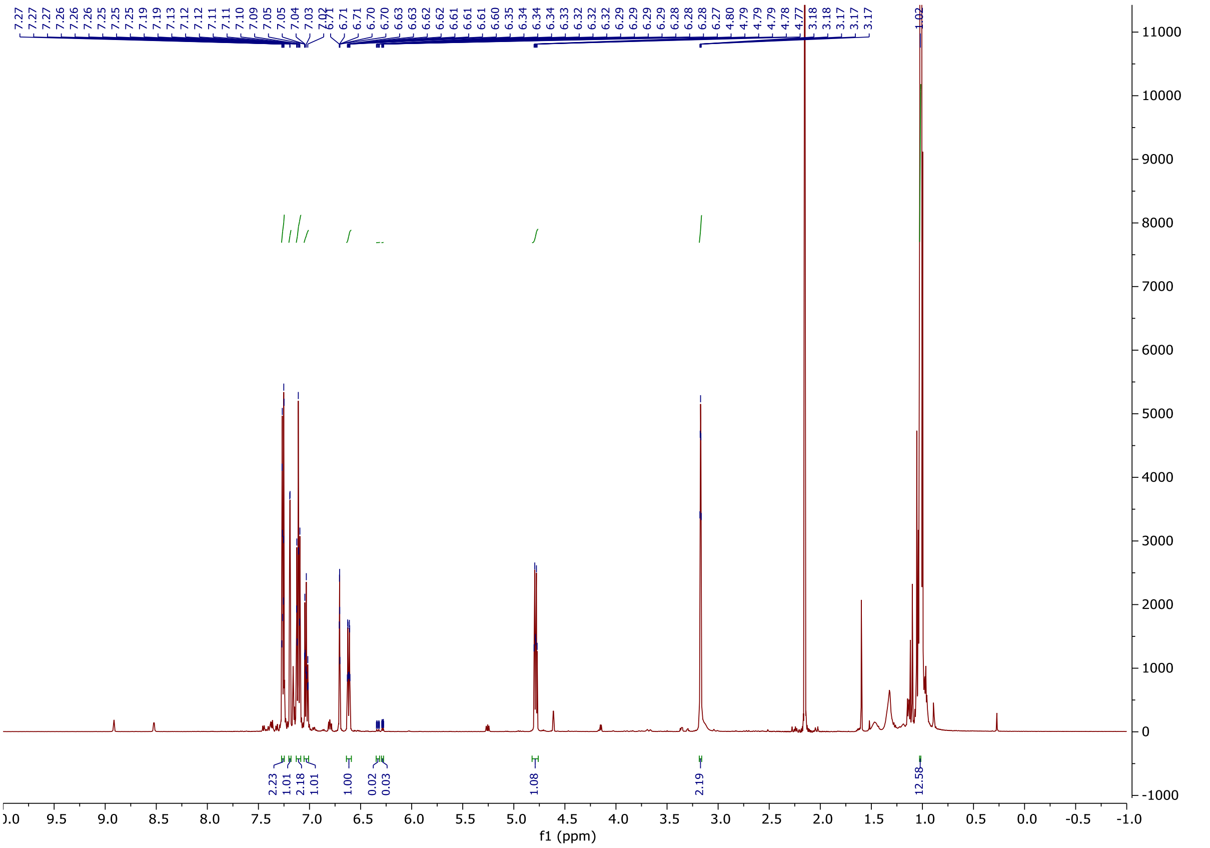

**Figure S23. $^{1}$H NMR spectrum of Hydroboration of 6f in $C_6D_6$.**

Hydroboration of 3,5-lutidine (**6g**):

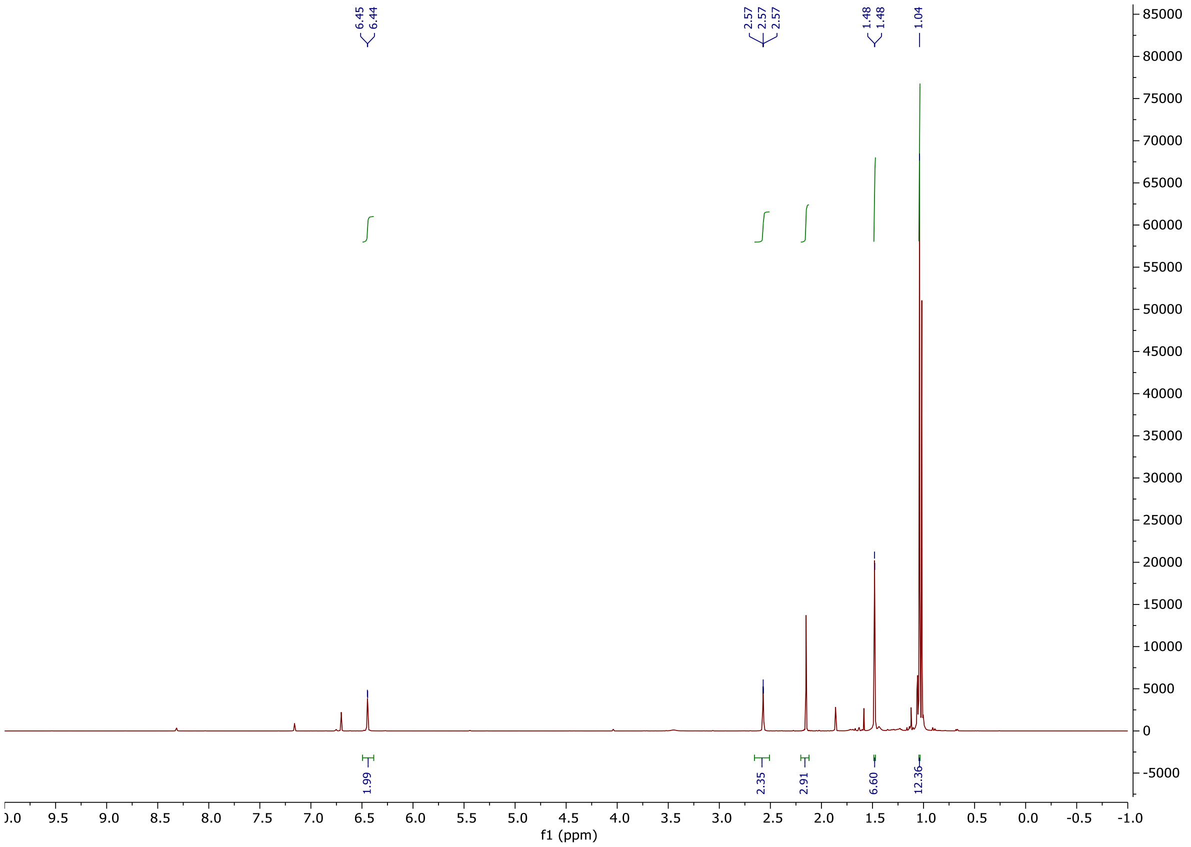


**Figure S24. $^{1}$H NMR spectrum of Hydroboration of 6g in $C_6D_6$.**

# Hydroboration of 1-methylbenzimidazole (**6h**):

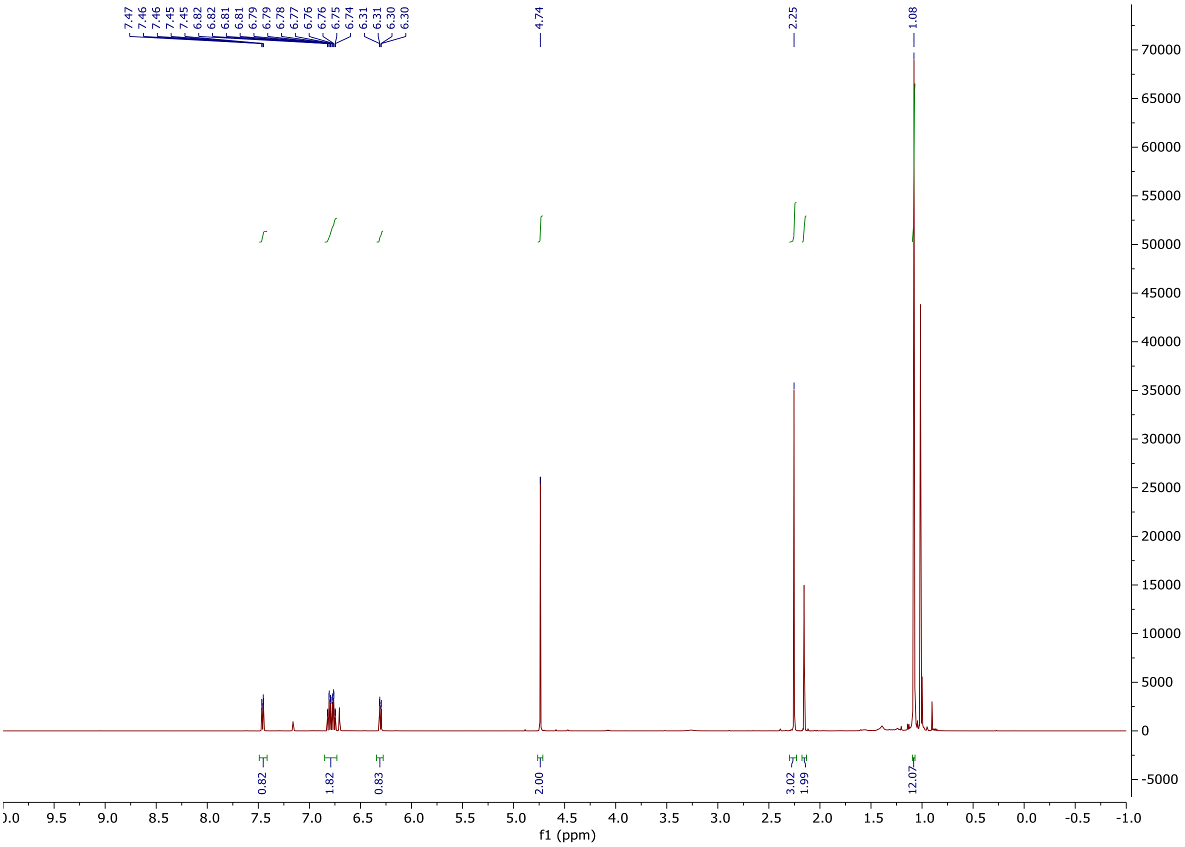


**Figure S25. $^1$H NMR spectrum of Hydroboration of 6h in $C_6D_6$.**

Hydroboration of methyl 3-pyridinecarboxylate (**6i**):

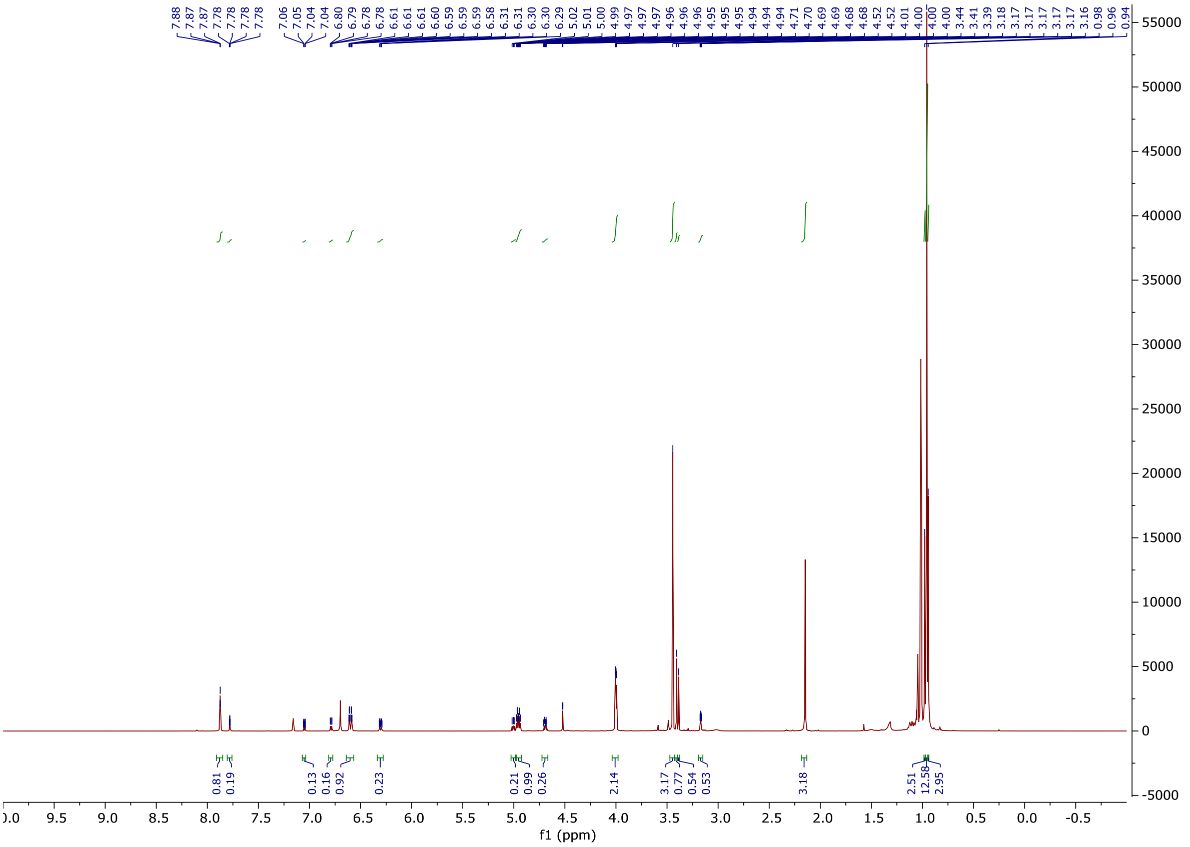

**Figure S26. $^1$H NMR spectrum of Hydroboration of 6i in $C_6D_6$.**

Hydroboration of 3-methoxypyridine (**6j**)

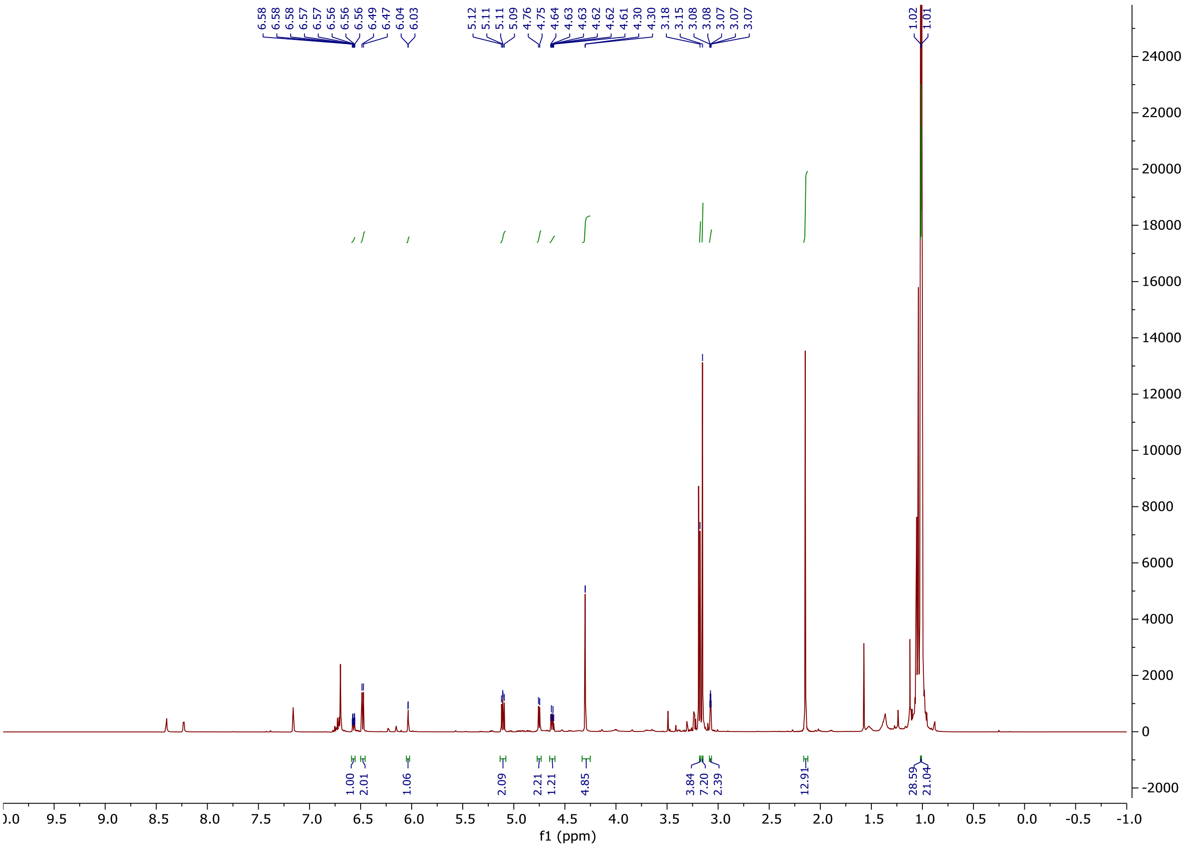


**Figure S27. $^{1}$H NMR spectrum of Hydroboration of 6j in $C_6D_6$.**

Hydroboration of methyl-3-pyridylketone (**6k**)

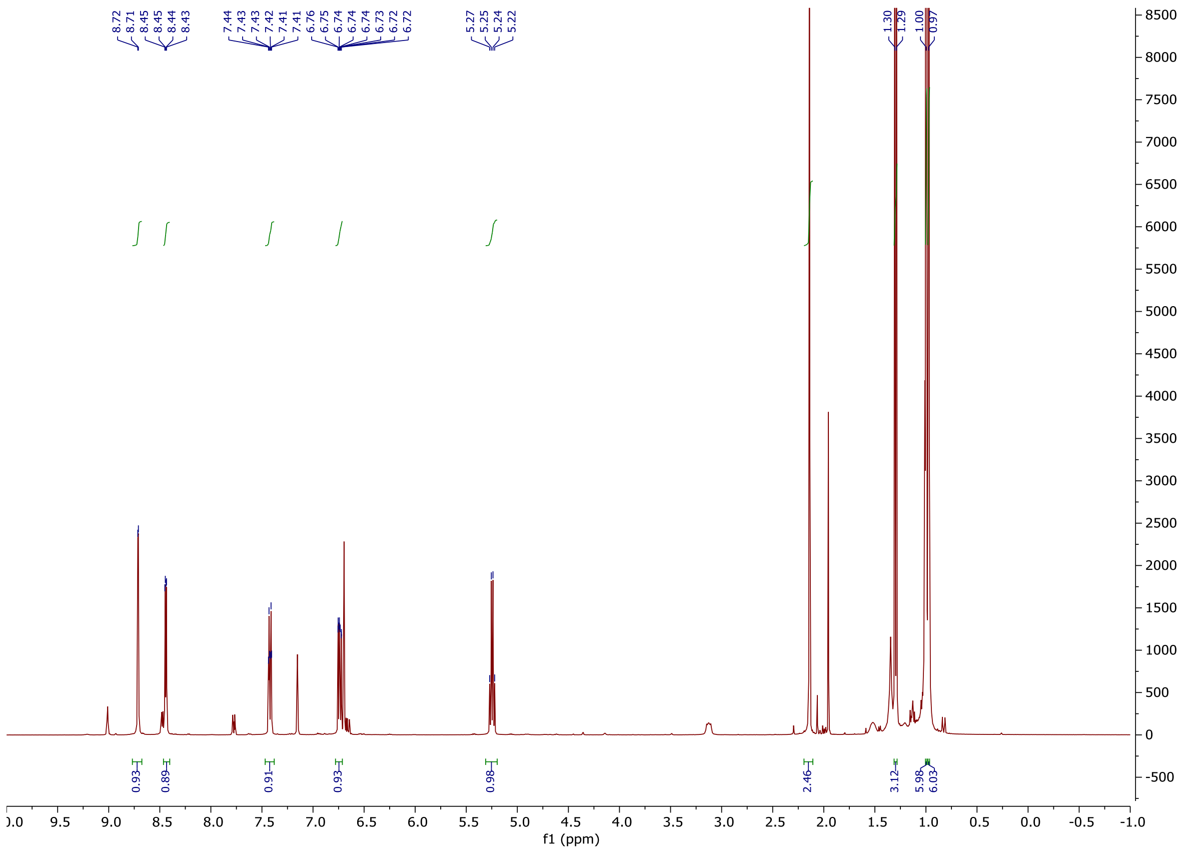


**Figure S28. $^{1}$H NMR spectrum of Hydroboration of 6k in $C_6D_6$.**

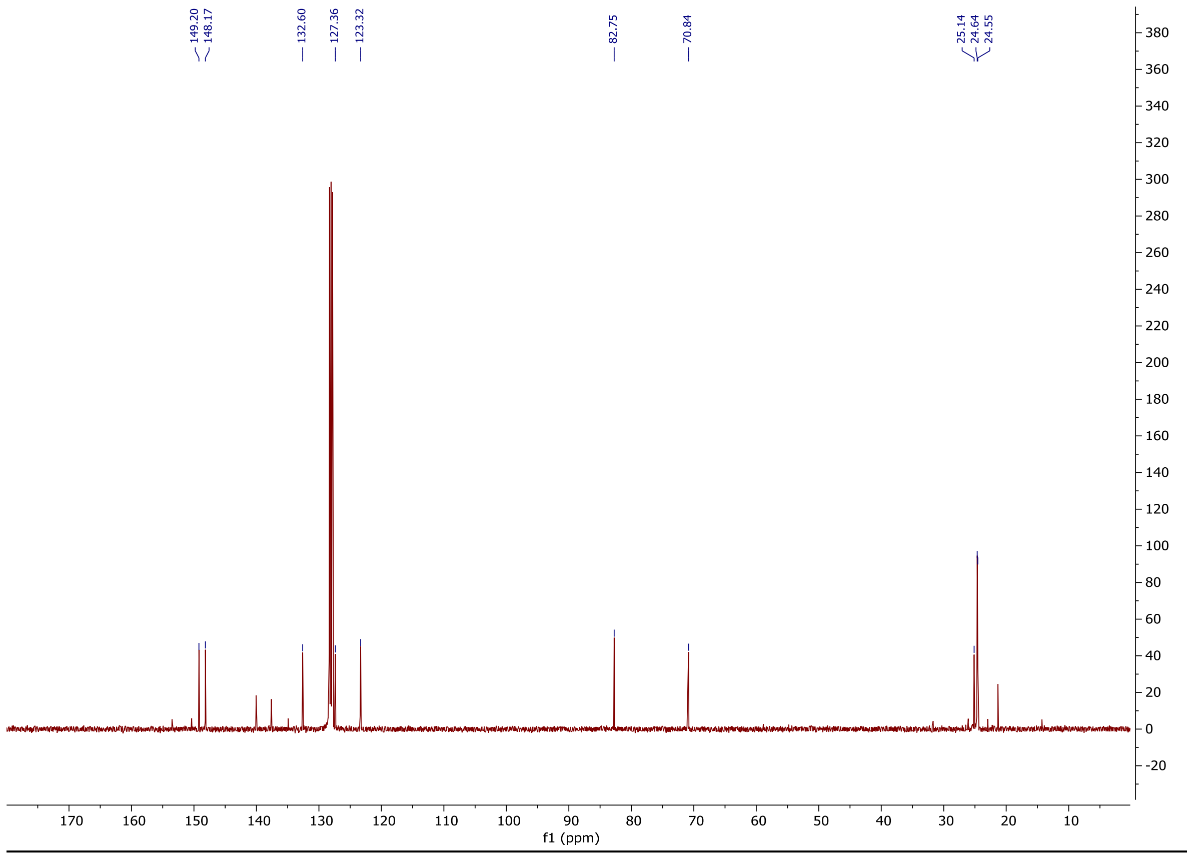


**Figure S29. $^{13}$C NMR spectrum of Hydroboration of 6k in $C_6D_6$.**

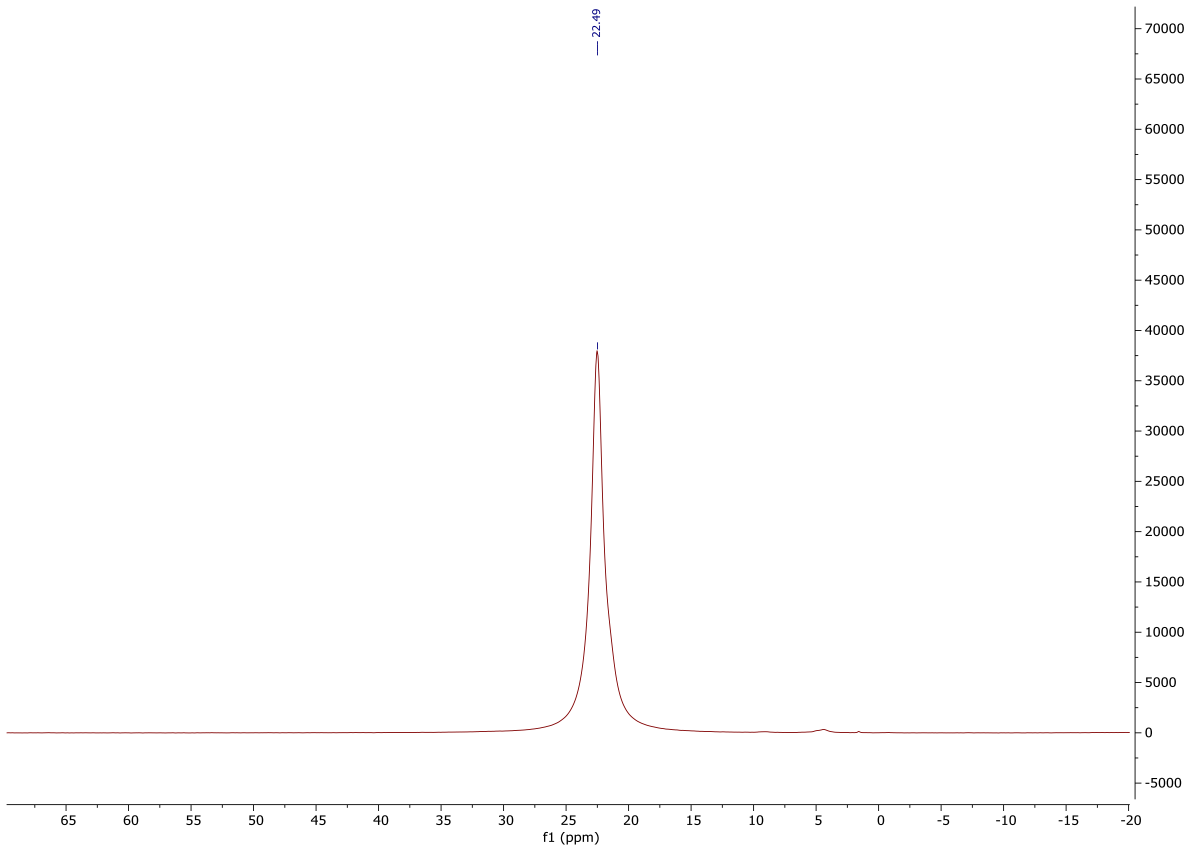


**Figure S30. $^{11}$B NMR spectrum of Hydroboration of 6k in $C_6D_6$.**

# Hydroboration of pyridine (**6a**) using **1**

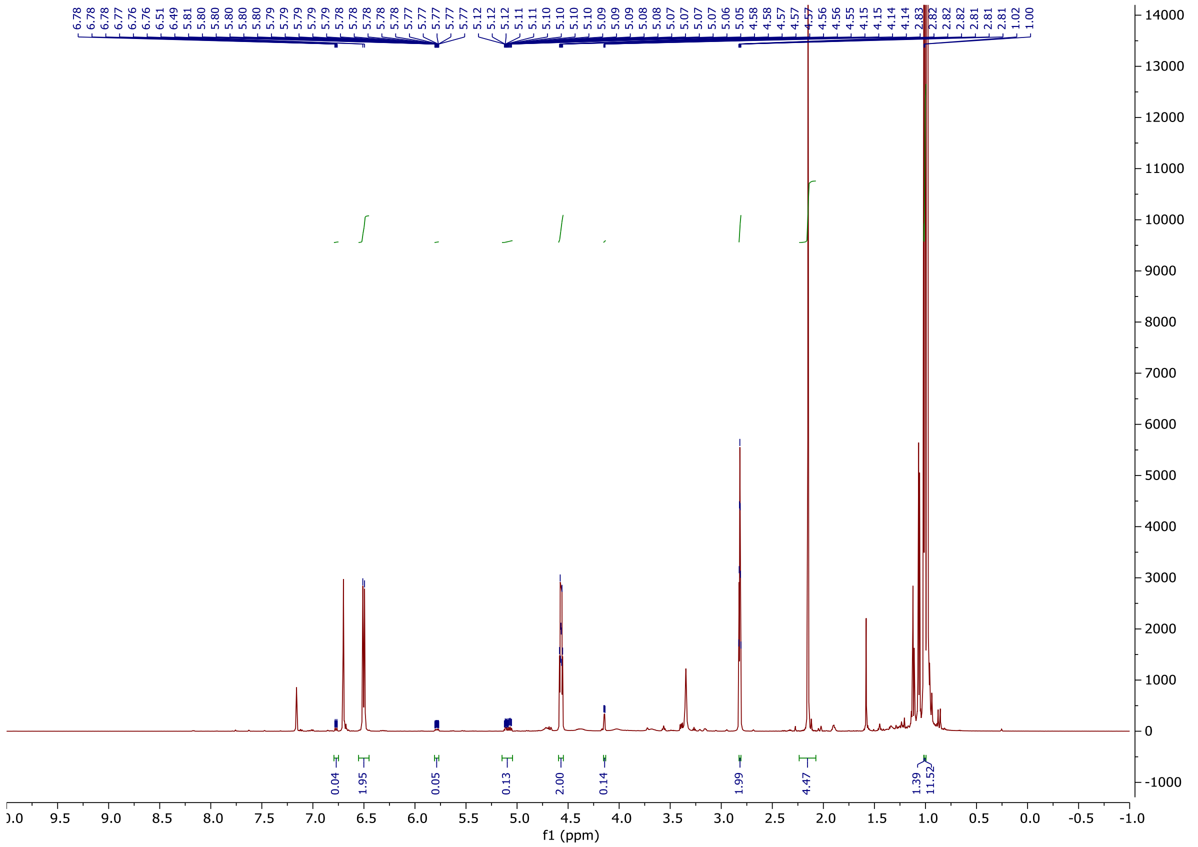

**Figure S31. $^{1}$H NMR spectrum of Hydroboration of 6a with 1 in $C_6D_6$.**

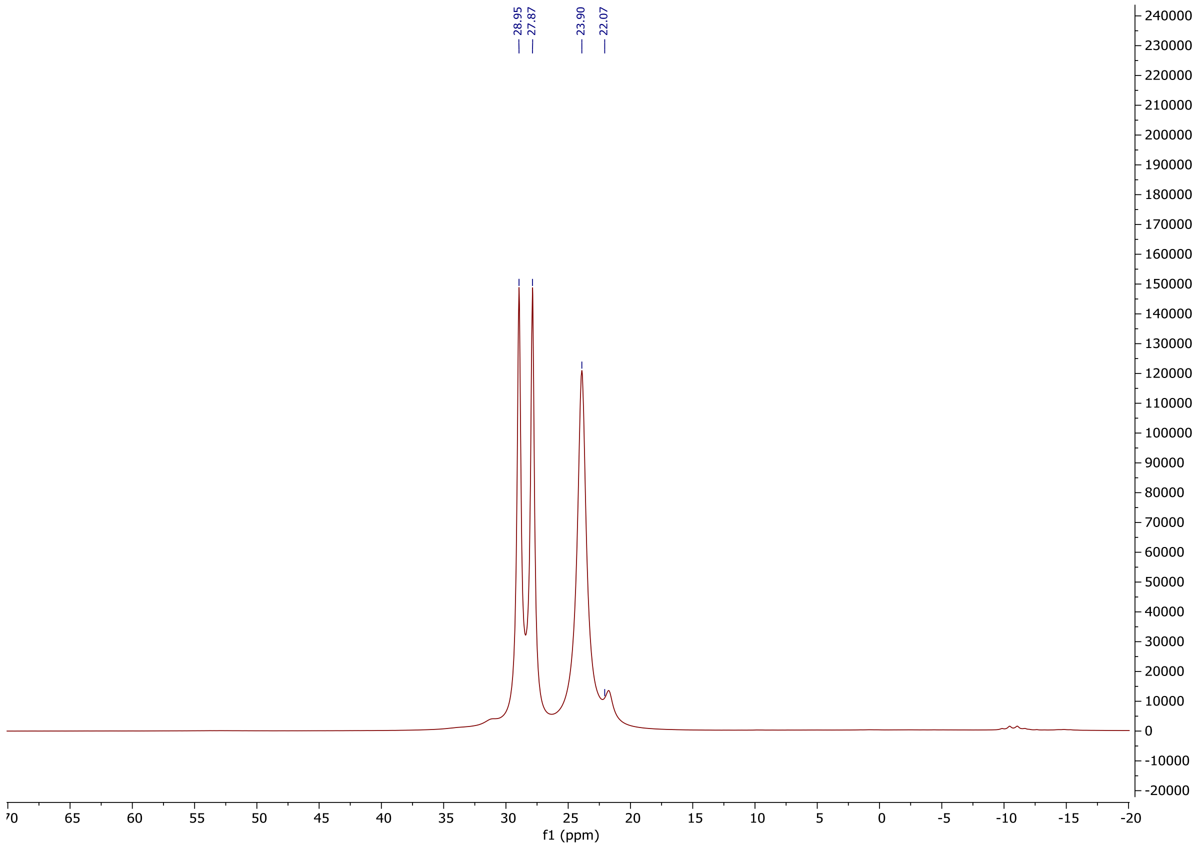

**Figure S32. $^{11}$B NMR spectrum of Hydroboration of 6a with 1 in $C_6D_6$.**

# 5.0 Infrared Spectra

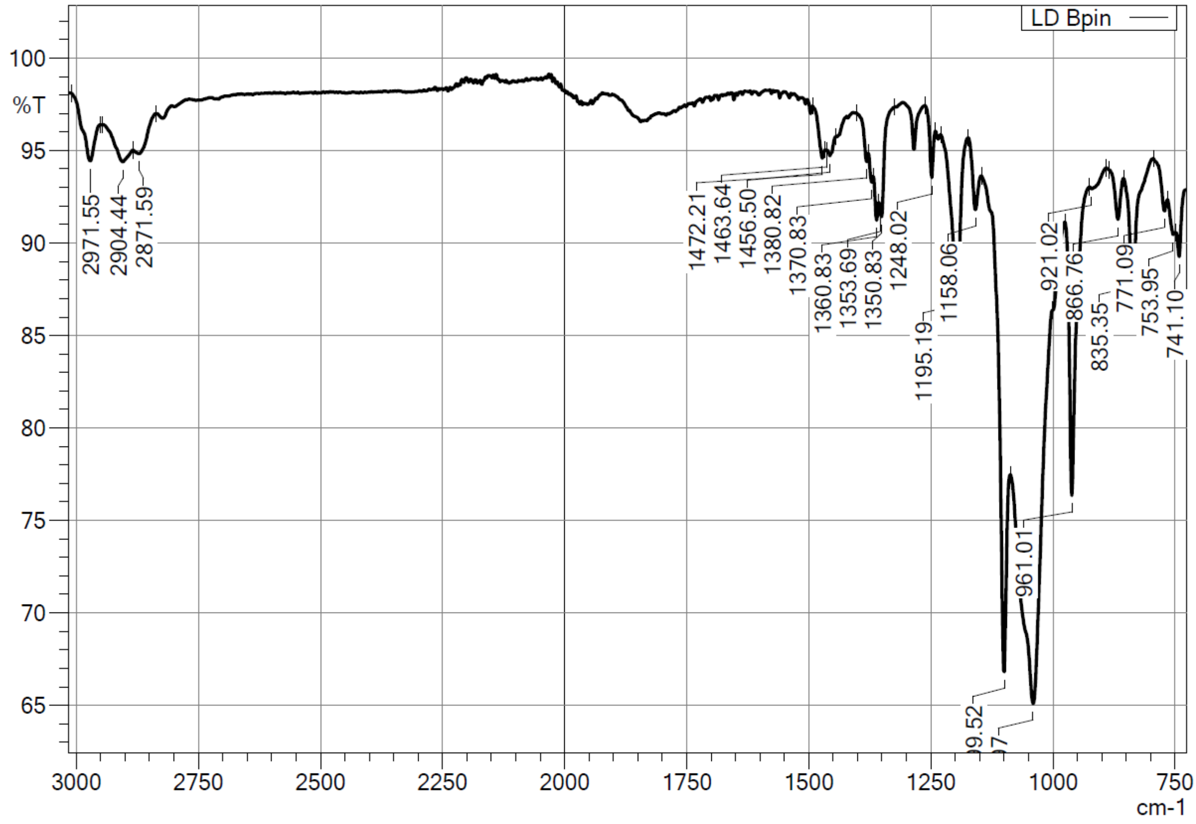


**Figure S33. IR spectrum of 1.**

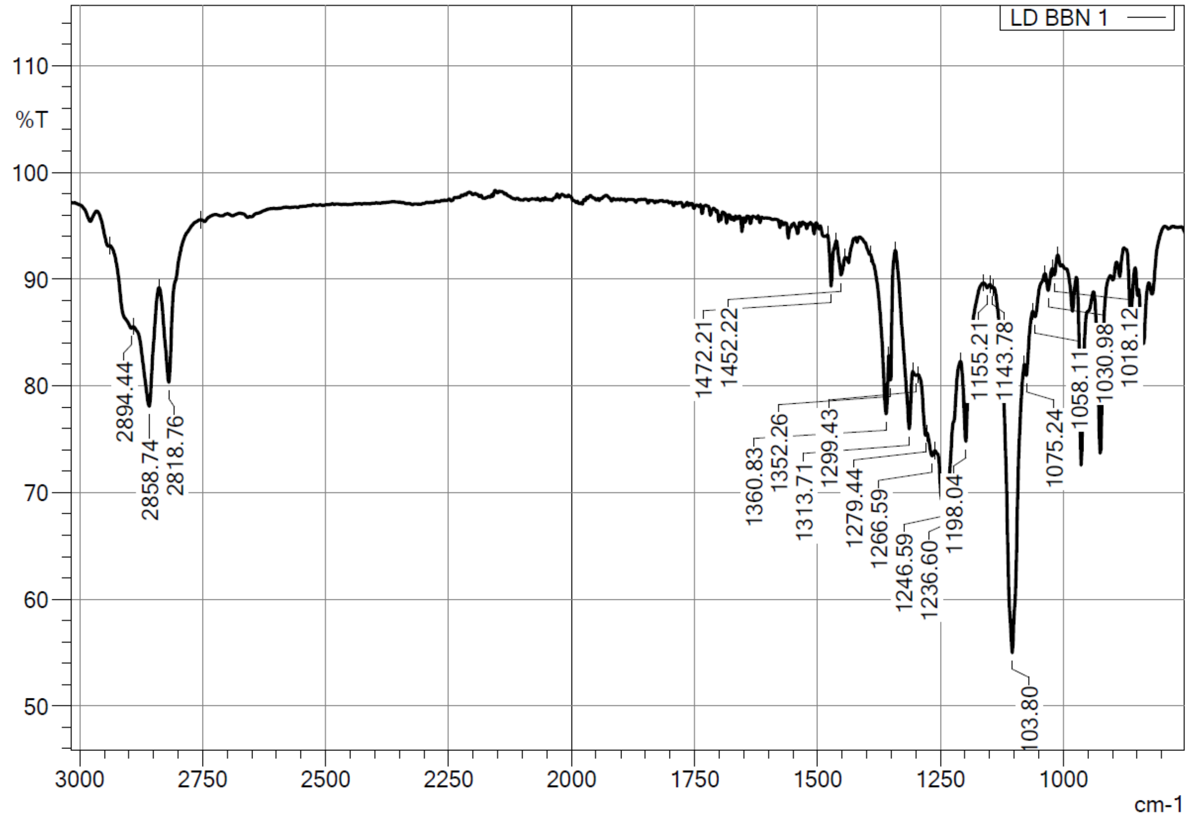


**Figure S34. IR spectrum of 4.**

**6.0 Crystallographic Details**

[K(DME)(18-c-6)][$ReH_7(Bpin)_3$] **1**: Refined as a 2-component twin. The twin law is

```
   Transforms h1.1(1)->h1.2(2)
   -0.42198 -0.57921 -0.04709
    1.41915 -0.42195  0.04692
    0.00113  0.00043  1.00004
```

The matrix corresponds to a rotation of 114.9 degrees about [001], so that the crystal is more properly described as a two-domain aggregate. Indexing was carried out with CELL_NOW. The diffraction images were integrated for both domains using two orientation matrices. The structure was solved and initially refined with the model of the anion consisting of [Re(BPin)3], that is without any hydrogens attached to the metal. All other H-atoms were placed on C in ideal positions, and all non-H atoms were modelled with anisotropic displacement parameters. The Re1 and B2 reside on a crystallographic .2. axis. The cation also has crystallographic two-fold symmetry. The refinement statistics were:

wR2 = 0.133622, GooF = S = 1.17321, Restrained GooF = 1.17321 for all data R1 = 0.056801 for 9034 Fo > 4sig(Fo) and 0.060189 for all 9318 data 242 parameters refined using 0 restraints. The adps were reasonably well-behaved, and when fitted to a TLS librational model (SHELXTL-XP) the residuals were 0.1184 and 0.1142 for the ligand based B1 and B2, respectively. The geometry about Re1 was:

```
Re1 B1     2.194(10)
Re1 B2     2.173(14)
B2 Re1 B1  118.6(2)
B2 Re1 B1' 118.6(2)
B1 Re1 B1' 122.9(5)
```

where B1' is the symmetry equivalent of B1 [-x+1, y, -z+1/2].

The final difference map contained several peaks within 1.15 Ang of Re1 with heights between 1.95 to 0.57 e/Ang**3; this is a common effect of residual absorption errors in heavy atom crystal structures. Several other peaks with heights between 1.50 and 0.80 e/Ang**3 were located at between 1.65 and 1.78 Ang from Re1, which are reasonable distances to hydride ligands. Three of these were assigned as H1-3 and could be refined provided the Re-H was restrained to 1.7 Ang. Other H-atoms could not be convincingly located.

Possible positions for the remaining H-atoms were calculated by DFT (see below). The optimised DFT model of the anion is given below in SHELX format with positions as fractional coordinates based on a metrically cubic unit cell with a = 15 Ang, space group P1.

```
TITL Converted
CELL 0.71073 15 15 15 90 90 90
ZERR 1 0 0 0 0 0 0
LATT -1
SFAC C H B O Re
UNIT 18 43 3 6 1
FVAR 1.00
Re1     5  0.468174  0.044662  0.235850  1.000000 0.05000
H1      2  0.524037  0.129235  0.168335  1.000000 0.06000
H2      2  0.456985 -0.058927  0.275916  1.000000 0.06000
H3      2  0.434675  0.151287  0.277438  1.000000 0.06000
H4      2  0.550133  0.051501  0.317875  1.000000 0.06000
H5      2  0.409193  0.033252  0.329360  1.000000 0.06000
H6      2  0.464340  0.433687  0.345160  1.000000 0.06000
H7      2  0.416840  0.324554  0.342262  1.000000 0.06000
H8      2  0.519235  0.342849  0.398444  1.000000 0.06000
H9      2  0.601327  0.467758  0.242182  1.000000 0.06000
H10     2  0.653086  0.391535  0.316663  1.000000 0.06000
H11     2  0.662597  0.375100  0.199597  1.000000 0.06000
H12     2  0.560360  0.416551  0.074065  1.000000 0.06000
H13     2  0.585504  0.300452  0.083373  1.000000 0.06000
H14     2  0.488496  0.335510  0.025536  1.000000 0.06000
H15     2  0.432060  0.481988  0.172989  1.000000 0.06000
H16     2  0.365211  0.411590  0.104959  1.000000 0.06000
H17     2  0.355810  0.405661  0.222864  1.000000 0.06000
B18     3  0.489155  0.197809  0.217909  1.000000 0.05000
C19     1  0.533247  0.340235  0.255544  1.000000 0.05000
C20     1  0.479596  0.362189  0.340019  1.000000 0.05000
C21     1  0.617577  0.397060  0.252824  1.000000 0.05000
C22     1  0.475087  0.343290  0.168272  1.000000 0.05000
C23     1  0.531446  0.349810  0.083055  1.000000 0.05000
C24     1  0.402850  0.414938  0.167691  1.000000 0.05000
O25     4  0.558183  0.247357  0.259751  1.000000 0.05000
O26     4  0.433034  0.256663  0.169123  1.000000 0.05000
H27     2  0.382888  0.062060  0.157016  1.000000 0.06000
H28     2  0.179809 -0.199400  0.306774  1.000000 0.06000
H29     2  0.292038 -0.159680  0.310686  1.000000 0.06000
H30     2  0.207218 -0.100994  0.367870  1.000000 0.06000
H31     2  0.064155 -0.110743  0.208888  1.000000 0.06000
H32     2  0.088672 -0.024107  0.286099  1.000000 0.06000
H33     2  0.097920 -0.004947  0.166603  1.000000 0.06000
H34     2  0.170027 -0.244414  0.132778  1.000000 0.06000
H35     2  0.267963 -0.242642  0.065995  1.000000 0.06000
H36     2  0.277507 -0.252965  0.183289  1.000000 0.06000
H37     2  0.135463 -0.098540  0.040151  1.000000 0.06000
H38     2  0.206325 -0.003527  0.056923  1.000000 0.06000
H39     2  0.245134 -0.096331 -0.004677  1.000000 0.06000
B40     3  0.344768 -0.017444  0.198220  1.000000 0.05000
C41     1  0.206132 -0.079444  0.225734  1.000000 0.05000
C42     1  0.244525 -0.119475  0.136796  1.000000 0.05000
```

| | | | | | | |
|---|---|---|---|---|---|---|
| C43 | 1 | 0.221851 | -0.139372 | 0.307231 | 1.000000 | 0.05000 |
| C44 | 1 | 0.107665 | -0.053078 | 0.221177 | 1.000000 | 0.05000 |
| C45 | 1 | 0.239465 | -0.220877 | 0.129824 | 1.000000 | 0.05000 |
| C46 | 1 | 0.204368 | -0.076668 | 0.052795 | 1.000000 | 0.05000 |
| O47 | 4 | 0.258529 | -0.000153 | 0.236714 | 1.000000 | 0.05000 |
| O48 | 4 | 0.336290 | -0.092834 | 0.142032 | 1.000000 | 0.05000 |
| H49 | 2 | 0.789766 | -0.143290 | 0.116631 | 1.000000 | 0.06000 |
| H50 | 2 | 0.671863 | -0.126348 | 0.117762 | 1.000000 | 0.06000 |
| H51 | 2 | 0.742301 | -0.044287 | 0.070604 | 1.000000 | 0.06000 |
| H52 | 2 | 0.888319 | -0.047744 | 0.226235 | 1.000000 | 0.06000 |
| H53 | 2 | 0.842058 | 0.043353 | 0.163174 | 1.000000 | 0.06000 |
| H54 | 2 | 0.832995 | 0.042933 | 0.283137 | 1.000000 | 0.06000 |
| H55 | 2 | 0.811176 | -0.211443 | 0.283299 | 1.000000 | 0.06000 |
| H56 | 2 | 0.715662 | -0.237150 | 0.348605 | 1.000000 | 0.06000 |
| H57 | 2 | 0.706367 | -0.232687 | 0.230900 | 1.000000 | 0.06000 |
| H58 | 2 | 0.820805 | -0.075882 | 0.394597 | 1.000000 | 0.06000 |
| H59 | 2 | 0.733118 | 0.005493 | 0.391943 | 1.000000 | 0.06000 |
| H60 | 2 | 0.714155 | -0.100625 | 0.439838 | 1.000000 | 0.06000 |
| B61 | 3 | 0.597000 | -0.011430 | 0.254041 | 1.000000 | 0.05000 |
| C62 | 1 | 0.742413 | -0.044162 | 0.214282 | 1.000000 | 0.05000 |
| C63 | 1 | 0.714743 | -0.103236 | 0.296260 | 1.000000 | 0.05000 |
| C64 | 1 | 0.736739 | -0.093311 | 0.124723 | 1.000000 | 0.05000 |
| C65 | 1 | 0.833458 | 0.001071 | 0.222799 | 1.000000 | 0.05000 |
| C66 | 1 | 0.738627 | -0.201826 | 0.288724 | 1.000000 | 0.05000 |
| C67 | 1 | 0.748648 | -0.065822 | 0.385519 | 1.000000 | 0.05000 |
| O68 | 4 | 0.676509 | 0.024807 | 0.215466 | 1.000000 | 0.05000 |
| O69 | 4 | 0.619528 | -0.093702 | 0.294844 | 1.000000 | 0.05000 |
| H70 | 2 | 0.497863 | -0.028292 | 0.155297 | 1.000000 | 0.06000 |

In this list the H-atoms attached to Re1 are H1-H5, H27 and H70. The geometry (in Angstrom and degrees) about Re1 is

Re1-B18 2.334 (a borohydride)
Re1-B40 2.148 (a borane)
Re1-B61 2.125 (a boryl)
B18-Re1-B40 120.85
B18-Re1-B61 106.37
B40-Re1-B61 130.22
The Re1-H distances span 1.667 to 1.827 Ang.

The anion deviates substantially from $C_2$ symmetry. If it were present in this crystal structure with the same geometry, disorder would be expected to be substantial and obvious in a Fourier map, but this is not observed. Nevertheless the positions of H4-H6 were inferred from the DFT result using the positions of Re1 and H1-3 to define the orientation of the DFT model. Thereafter H1-7 were allowed to ride on Re1 with Uiso(H) = 1.5Ueq(Re1). Only H2

has full occupancy, the others are disordered by the two-fold axis and have half-occupancy. The final refinement statistics are:

REM  mo_jl19002_0m_4 in C2/c
REM wR2 = 0.131599, GooF = S = 1.15474, Restrained GooF = 1.15474 for all data
REM R1 = 0.055955 for 9034 Fo > 4sig(Fo) and 0.059356 for all 9318 data
REM 243 parameters refined using 0 restraints

These figures represent only a tiny improvement over those obtained without any hydrides placed at all (see above, e.g. R1 = 0.056801).  The identity of the anion in this structure is thus not established with much certainty by the crystal structure.  Although the presence of some hydride density was evident in a difference map, it is also necessary to assign the oxidation state of Re (+7) on the basis of the colour of the crystal (colourless) and the interpretation of the proton NMR spectrum in terms of seven fluxional hydride ligands.

In addition to the disagreement between the crystal and DFT structures, in the B-Re-B angles, there are also differences in the Re-B bond distances. Overall, the experimental distances and angles are similar to the average values of the DFT model [distances, expt: 2.194(10) and 2.173(14) Ang, DFT average: 2.202 Ang, angles, expt 120 deg, DFT 119.1 deg]. It is possible that the anion is completely disordered, such that all three ligands and the hydrides are scrambled about the metal, and that the geometrical parameters seen crystallographically are averages.  A rather complex refinement model was constructed in which each site was occupied by one Bpin ligand with a Re-B distance of 2.334 Ang, and other with a Re-B distance equal to 2.1365 Ang (the average of the the shorter Re-B distances in the DFT model). The hydrides were again placed using the DFT model. The refinement statistics were:

REM  mo_jl19002_0m_4 in C2/c
REM wR2 = 0.130242, GooF = S = 1.16066, Restrained GooF = 1.14558 for all data
REM R1 = 0.055962 for 9034 Fo > 4sig(Fo) and 0.059352 for all 9318 data
REM 282 parameters refined using 310 restraints

These are essentially identical to the model described above, but with a larger number of parameters.

[K(DME)(18-c-6)][Re($\kappa_2$-$H_2$BBN)$_4$] **4**: A molecule of DME coordinated to K1 (C63, O7, C64, C65, C65A, O8, O8A, C67) was positionally disordered over two positions. As a result, C65/65A were refined with an occupany ratio of 0.734:0.266, and O8/8A was refined with an

occupancy ratio of 0.720:0.280. The hydrides in the anion were placed in positions based on the difference synthesis and refined with free uiso. Bond lengths between C65, C64, and C65A were restrained to be equal within the standard uncertainty of 0.02 through the use of SADI command. Data collected at 220 K due to suspected phase transition upon cooling.

| Complex | [K(DME)(18-c-6)][ReH$_4$(Bpin)($\eta^2$-HBpin)($\kappa^2$-H$_2$Bpin)] **1** | [K(DME)(18-c-6)][Re($\kappa_2$-H$_2$BBN)$_4$] **4** |
|---|---|---|
| Chemical formula | $C_{34}H_{77}B_3O_{14}KRe$ | $C_{48}H_{98.03}B_4KO_8Re$ |
| Mr | 967.68 | 1071.83 |
| Crystal system, space group | C2/c | P -1 |
| Temperature (K) | 100 | 220 |
| a, b, c (Å) | a=14.7681(15) b=23.105(2) c=13.7109(14) | a=11.2627(2) b=15.9727(3) c=16.0123(2) |
| α, β, γ (°) | 90, 91.809(3), 90 | 96.714(1), 91.290(1), 102.351(2) |
| V (Å$^3$ ) | 4676.1(8) | 2791.22(8) |
| Z | 4 | 2 |
| Radiation type | Mo Kα | Mo Kα |
| μ (mm$^{-1}$) | 2.743 | 2.296 |
| Crystal size (mm) | 0.578 × 0.059 × 0.053 | 0.354 × 0.203 × 0.083 |
| Diffractometer | Bruker D8 VENTURE | Rigaku Oxford Diffraction Xcalibur |
| Absorption correction | Multi-scan, TWINABS-2012/1 (Bruker,2012) | Analytical *CrysAlis PRO* 1.171.38.42b (Rigaku Oxford Diffraction, 2015) |
| Tmin, Tmax | 0.322, 0.430 | 0.942, 0.980 |
| θmin, θmax | 2.24, 26.430 | 3.6110, 30.525 |
| No. of measured, | 158838, 9318, 9034 | 73081, 15734, 14293 |

| | | |
|---|---|---|
| independent and observed [I > 2σ(I)] reflections | | |
| Rint | 0.0709 | 0.0333 |
| R[$F^2$ > 2σ($F^2$)], wR($F^2$), S | 0.0560, 0.1316, 1.155 | 0.0227, 0.0461, 1.039 |
| No. of reflections | 9312 | 73081 |
| No. of parameters | 243 | 613 |
| No. of restraints | 0 | 1 |
| Δρmax, Δρmin (e $Å^{-3}$) | 1.93, -2.50 | 0.74, -0.70 |
| CCDC Code | 1979242 | 1979241 |

**Table S35. Table of crystallographic data for solid-state structures of 1 and 4.**

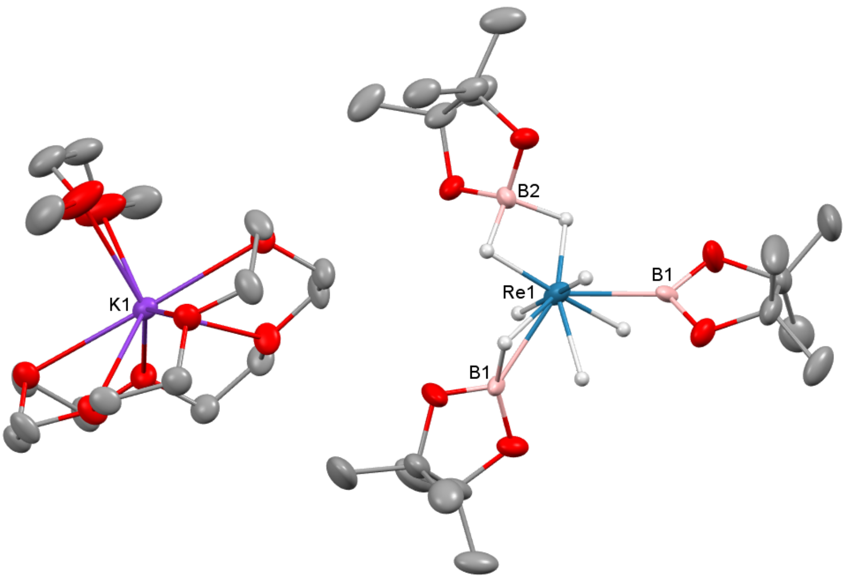


**Figure S36. Solid-state structure of 1. For clarity, all hydrogen atoms (except hydrides) are omitted; displacement ellipsoids are drawn at 50% probability. Selected distances (Å): Re1-B1 2.198(10); Re1-B2 2.174(14). Selected angles (°): B2-Re1-B1 118.6(2); B1-Re1-B1 122.9(5).**

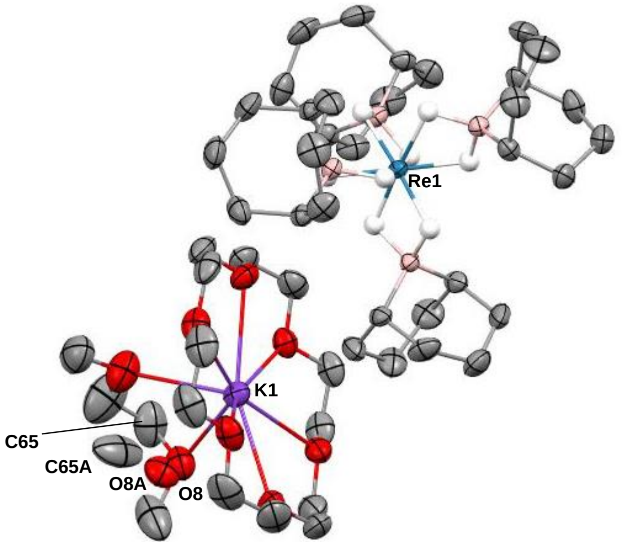


**Figure S37. Solid-state structure of 4. For clarity, all hydrogen atoms (except hydrides) are omitted; displacement ellipsoids are drawn at 50% probability. O8 and C65 were modelled anisotropically over two positions due to positional disorder (labelled as O8 and O8A, C65 and C65A respectively). Selected distances (Å): B-H (avg.) 1.284(2); Re-B (avg.) 2.296(2); Re-H (avg.) 1.731(2). Selected angles (°): H-B-H (avg.) 94.8(3); H-Re-H (avg.) 66.23 (3).**

## 7.0 Geometry optimisation of the [$ReH_7(Bpin)_3$] anion

The seven hydrogen atoms around the Re centre in [$ReH_7(Bpin)_3$]$^-$ were located in a non-biased way through a series of AIRSS/CASTEP geometry optimisation calculations.[11, 12] The model comprised the anion located inside a 15 Å$^3$ cubic box alongside a non-coordinating $K^+$ to maintain charge neutrality of the periodic boundary condition box. AIRSS constructed the initial models by positioning the hydrogen atoms randomly on a sphere of radius 1.6-1.85 Å centred on the Re coordinate. Two further constraints were imposed on the initial hydrogen atom placement, to ensure a minimum separation of 1.1 Å between the hydrogen atoms (to prevent possible formation of $H_2$) and 1.5 Å between the hydrogen atoms and the potassium centre (to prevent loss of hydrogen around the Re centre through the formation of K-H bonds). Over 30 random input structures were generated in this way, and optimised using CASTEP (version 16) for full atom optimisation (basis set energy cut-off: 750 eV; functional: PBE; dispersion correction: TS; pseudopotentials: on-the-fly; geometry optimisation criteria: energy tolerance: $5 \times 10^{-4}$ eV, maximum force tolerance: $5 \times 10^{-2}$ eV Å$^{-1}$ atomic displacement tolerance: $5 \times 10^{-2}$ Å). The relative energies of the resulting optimised structures are arranged, from lowest to highest, in Figure S36 below, alongside images of the minima obtained.

(a)

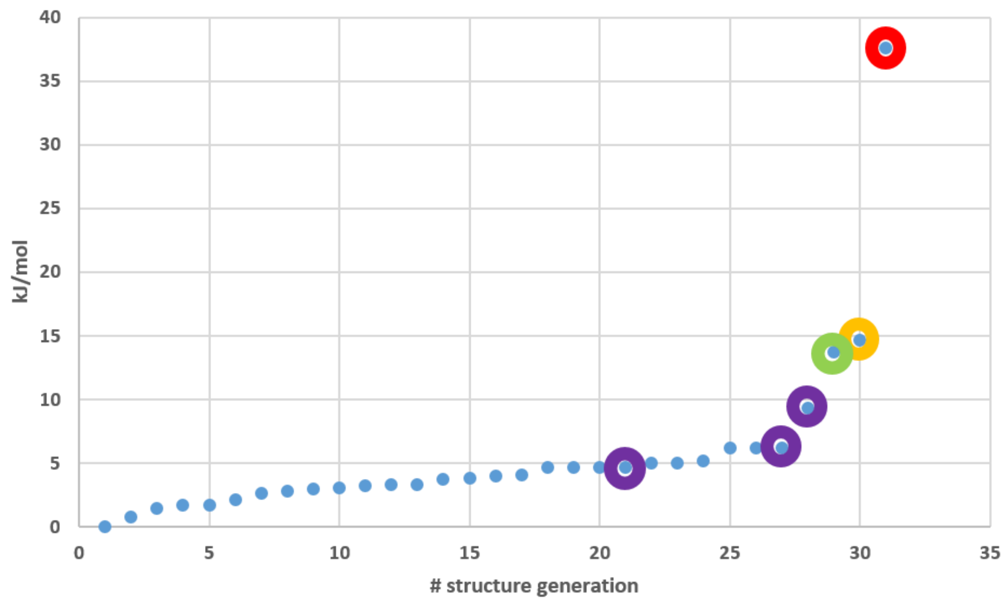


(b)

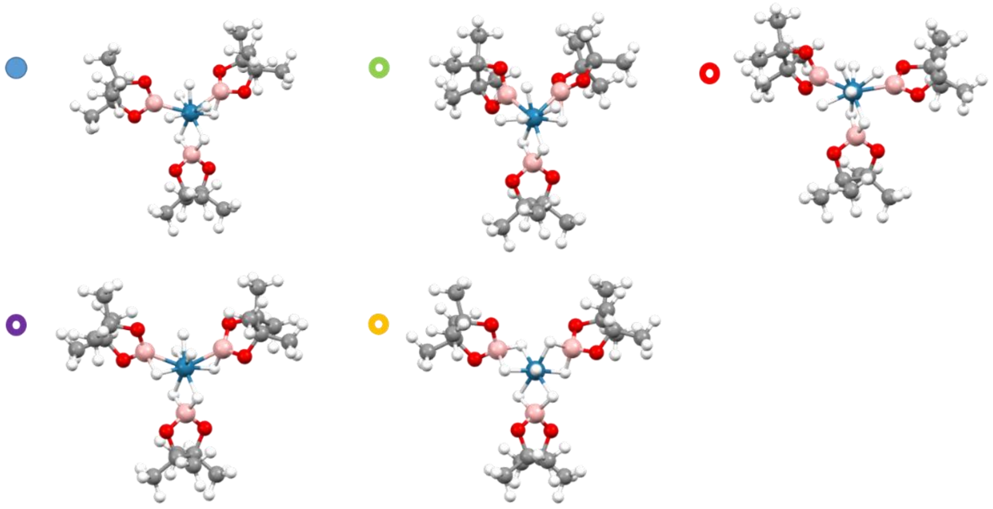

**Figure S38 (a) Relative energies of the optimised structures of $[ReH_7(Bpin)_3]^-$, generated through randomising the initial positions of the seven hydrogen atoms around the Re centre, and (b) the five minima obtained.**

The first point to observe is that all (bar six) of the optimised structures obtained conform to one structure type, where the Re is coordinated by one bridging dihydroborate ligand and one

σ-borane ligand, leaving four Re-H (three equatorial, one axial) terminal interactions, and thereby creating a Re(V) centre. The repetition of this basic structural motif, which falls over an energy range of no more than 6 kJ $mol^{-1}$, present variations in the three B–B–B angles (which typically present as ca. 110, 115 and 130 ± 5°). The next most stable minimum, which was found three times (some 5-10 kJ $mol^{-1}$ above the lowest energy structure) comprises one bridging dihydroborate ligand and two σ-borane ligands, leaving three (equatorial) Re-H bonds; the biggest variations between these structures again rests with the B–B–B angles, which adopt similar ranges to that noted above. The three remaining highest energy structures each have a multiplicity of one. The first (at +14 kJ $mol^{-1}$) is very similar to the lowest energy motif, except that the four terminal Re-H bonds now adopt a two equatorial, two axial formation; the second (at +15 kJ $mol^{-1}$) comprises three bridging dihydroborate ligands and one axial Re-H bond. The final highest energy structure (at +38 kJ $mol^{-1}$) has just one bridging dihydroborate and five terminal Re-H (three equatorial, two axial) bonds, and thereby represents a Re(VII) centre.

The most likely reason for the variation in energy and geometry for the multiple instances of the lowest energy motif is the relatively low quality convergence criteria used in the AIRSS process. Taking three of the minima obtained from the above run, and re-optimising to more stringent conditions (energy tolerance: $2 \times 10^{-6}$ eV, maximum force tolerance: $1 \times 10^{-2}$ eV $Å^{-1}$ atomic displacement tolerance: $5 \times 10^{-4}$ Å) has clearly resulted in the same minimum being obtained, as witnessed by the structural overlay diagram shown in Figure S37. The three B–B–B angles have now converged to 108.6, 116.9 and 133.0°. The energy range of ca. 5 kJ $mol^{-1}$ still remains, however. In all likelihood this is due to some small variation in interaction energy from the $K^+$ counterion; variations in its location are apparent from the overlay diagram shown below.

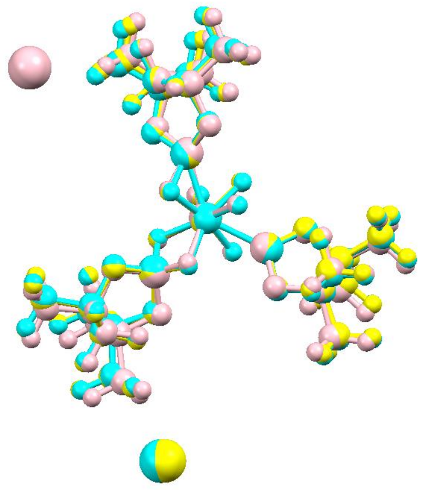

**Figure S39. Overlay of the optimised structures obtained for three of the lowest energy structures in the AIRSS run, using tighter optimisation criteria.**

## 8.0 References


1. Morris, D. S.; Weetman, C.; Wennmacher, J. T. C.; Cokoja, M.; Drees, M.; Kühn, F. E.; Love, J. B., Reduction of carbon dioxide and organic carbonyls by hydrosilanes catalysed by the perrhenate anion. *Catalysis Science & Technology* **2017,** *7* (13), 2838-2845.
2. Sheldrick, G., SHELXT - Integrated space-group and crystal-structure determination. *Acta Crystallographica Section A* **2015,** *71* (1), 3-8.
3. Sheldrick, G., Crystal structure refinement with SHELXL. *Acta Crystallographica Section C* **2015,** *71* (1), 3-8.
4. Dolomanov, O. V.; Bourhis, L. J.; Gildea, R. J.; Howard, J. A. K.; Puschmann, H., OLEX2: a complete structure solution, refinement and analysis program. *Journal of Applied Crystallography* **2009,** *42* (2), 339-341.
5. Arrowsmith, M.; Hill, M. S.; Hadlington, T.; Kociok-Köhn, G.; Weetman, C., Magnesium-Catalyzed Hydroboration of Pyridines. *Organometallics* **2011,** *30* (21), 5556-5559.
6. Intemann, J.; Lutz, M.; Harder, S., Multinuclear Magnesium Hydride Clusters: Selective Reduction and Catalytic Hydroboration of Pyridines. *Organometallics* **2014,** *33* (20), 5722-5729.
7. Zhang, F.; Song, H.; Zhuang, X.; Tung, C.-H.; Wang, W., Iron-Catalyzed 1,2-Selective Hydroboration of N-Heteroarenes. *Journal of the American Chemical Society* **2017,** *139* (49), 17775-17778.
8. Fan, X.; Zheng, J.; Li, Z. H.; Wang, H., Organoborane Catalyzed Regioselective 1,4-Hydroboration of Pyridines. *Journal of the American Chemical Society* **2015,** *137* (15), 4916-4919.
9. Kaithal, A.; Chatterjee, B.; Gunanathan, C., Ruthenium-Catalyzed Regioselective 1,4-Hydroboration of Pyridines. *Organic Letters* **2016,** *18* (14), 3402-3405.

11. Ab initio random structure searching, C. J. Pickard and R. J. Needs, *J. Phys. Condens. Matter* **2011**, *23*, 053201.

12. First principles methods using CASTEP, Zeitschrift fuer Kristallographie 220(5-6) pp. 567-570 (2005), S. J. Clark, M. D. Segall, C. J. Pickard, P. J. Hasnip, M. J. Probert, K. Refson and M. C. Payne.
